# Mesocosm Study of Chemical Treatments on Methane Emissions in Oil Sands Tailings Ponds—Part I: Focusing on the Change of Microbial Communities and Tailings Dewaterability

**DOI:** 10.1021/acsomega.5c07316

**Published:** 2025-10-13

**Authors:** Xiaomeng Wang, Nayereh Saborimanesh, Petr Kuznetsov, Amanda Cook, Jordan Elias, Louis Jugnia, Bipro Ranjan Dhar, Ania Ulrich

**Affiliations:** † Natural Resources Canada, CanmetENERGY Devon, 1 Oil Patch Drive, Devon, Alberta T9G 1A8, Canada; ‡ Faculty of Engineering, Civil and Environmental Engineering Department, 3158 University of Alberta, 9211 116 Street, Edmonton, Alberta T6G 2H5, Canada; § Energy, Mining and Environment Research Centre, 6356 National Research Council Canada, 6100 Royalmount Avenue, Montreal, Quebec H4P 2R2, Canada

## Abstract

In this study, we proposed mitigation strategies to reduce methane emissions from oil sands tailings ponds and determined the extent to which certain chemicals (Na_2_MoO_4_·2H_2_O, Fe_2_(SO_4_)_3_, Na_2_SO_4_, and Na_3_C_6_H_5_O_7_·2H_2_O) could affect the methanogenesis process. Lab-scale mesocosms were used to compare the amount of fugitive emissions between paraffinic and naphthenic producer tailings. The inter-relationships among different parameters, such as methane, water chemistry, residual bitumen content in tailings, and microbial community, were investigated before and after the methane inhibition process. It was found that under different chemical treatment regimens, methanogenic populations were either suppressed or stimulated, demonstrating that functionally similar disturbances in natural systems may result in distinct responses of the microbial populations involved. The 16S RNA gene sequencing data revealed that both solvents and chemical treatments significantly impacted microbial diversity and communities in tailings, leading to notable shifts in dominant microbial families and a decrease in diversity in the treated samples. These treatments affected methanogenic families, reducing the abundance of archaeal methanogens (e.g., *Methanegulaceae*) while increasing the presence of microbial families involved in hydrocarbon degradation, such as *Spirochaetaceae* and *Thermovirgaceae*. This study lays the groundwork for potential economically viable approaches to reduce methane emissions from oil sands tailings ponds.

## Graphical abstract

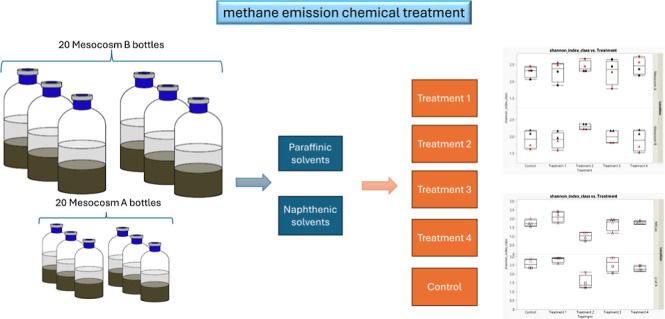

## Introduction

Degradation of air quality due to oil sands operations is one of the largest concerns for stakeholders and regulators. One contributor to poor air quality can be methane (CH_4_) released from tailings ponds. The CH_4_ emissions from tailings ponds accounted for 45% of the total CH_4_ emissions from oil sands facilities.^1^ For example, biogenic methane emissions from the Mildred Lake Settling Basin alone have been estimated at 43,000 m^3^/day.^2^ Another study estimated that methane production contributes 60–80% of gas flux over oil sands tailings ponds, with a daily emission of up to 100 million L of CH_4_ from one pond.^3^ Given that methane is about 25 to 30 times more potent than CO_2_ as a greenhouse gas, reducing CH_4_ emission from tailings ponds would have a significant impact on lowering the overall emissions from a mining operation. Nevertheless, challenges associated with oil sands tailings ponds include not only the emission of biogenic greenhouse gases (GHGs) (such as CH_4_ and CO_2_) but also the presence of inorganic and organic contaminants (metals, salts, petroleum hydrocarbons, naphthenic acids, etc.) and very slow consolidation of fine tailings solids.

Reducing fugitive emissions from oil sands tailings ponds requires a two-pronged approach, in which the first and foremost method is to reduce the amount of solvent entering tailings ponds by optimizing solvent recovery.^4^ The second method would be to inhibit the methanogenesis of solvent already in the ponds.^5^ Solvent recovery would reduce greenhouse gas (GHG) emissions in the long run, but the amount of solvent in tailings ponds today is already sufficient to drive substantial methanogenesis for years to come. Thus, the second method is needed in the meantime to achieve both an immediate effect and a long-term effect on reducing GHG emissions from tailings ponds. The approach will also be beneficial for both the environment and industry if it is applied on a commercial scale.

It appears that the type of solvent in the tailings ponds may affect the GHG emissions. Currently, Syncrude, Suncor, and Canadian Natural Resources Ltd. use a naphthenic diluent for the bitumen froth treatment process, whereas Shell Albian and Imperial Oil use a paraffinic diluent.^6^ Therefore, the tailings ponds produced by different oil sands operators will house different types of residual solvents. An industry report shows that the only producers with significant reported methane emissions are Suncor and Syncrude, both of which use a naphthenic diluent.^7^ However, the theoretical modeling of GHG emissions from tailings ponds conducted by Alberta Environment and Sustainable Resource Development (AESRD) demonstrates that equivalent amounts of GHG emissions can be produced by tailings ponds of both paraffinic and naphthenic froth treatment operators.^8^ Thus, further research is needed to investigate the underlying causes of methane emissions from tailings ponds across different froth treatment operations.

In the past, various research groups have been searching for effective chemical or biological treatments to inhibit methanogenesis in oil sands tailings. For example, Allam et al. reported that treatment with 3% protease, 3% lysozyme, and 5000 ppm lime reduced CH_4_ production by 52%, 28%, and 25%, respectively, in oil sands tailings, and these reductions were weakly associated with archaeal abundance.^5^ Although enzyme treatment resulted in a greater reduction in CH_4_ production compared with lime treatment, it also exhibited higher toxicity. Nevertheless, the high cost of enzymes and the large dosage required for lime both may limit their feasibility for industrial-scale application in oil sands tailings ponds. In addition, it is recognized that consuming labile hydrocarbons under nitrate-, iron-, and sulfate-reducing conditions is potentially a promising strategy for GHG mitigation in oil sands tailings.^9^ Methanogenesis was inhibited in laboratory incubations by nearly 50% when sulfate was supplied at pond-level concentrations, suggesting that in situ sulfate reduction can substantially minimize methane emissions.^10^ However, the synergy of these reducing conditions on methane production inhibition has not been examined. Also, it has long been recognized that the sulfate reduction and methanogenesis are both inhibited in the presence of 20 mM molybdate.^11^ Thus, in this study, we were particularly interested in the impact of sulfate-reducing conditions on methanogenesis inhibition by examining the synergy effects between sulfate and other chemicals, such as iron and molybdate.

The ultimate goals of this study are as follows: (i) to provide a comprehensive understanding of how various chemical additions impact methane production, water chemistry, microbial communities, and tailings consolidation in tailings ponds in order to assess the overall effect of modifying the natural methanogenesis process and (ii) to examine how solvents and the origin of the tailings influence the outcomes of chemical treatments.

## Experimental Section

### Materials

Two types of fluid fine tailings (FFT) sampled from the oil sands region in northern Alberta were incorporated into separate mesocosms, referred to as Mesocosm A samples and Mesocosm B samples, respectively. Mesocosm A samples were enriched with a mixed culture of methanogens, while Mesocosm B samples only contain indigenous methanogens from oil sands tailings. The paraffinic solvent (a mixture of 40% isopentane and 60% *n*-pentane by weight) and naphthenic solvent (a mixture of 40% toluene and 60% heptane by weight) were prepared in the lab, simulating different froth treatment processes, and were subsequently used to initiate the methane production in the mesocosms. These solvents were purchased from Fisher Scientific. Three chemicals including Na_2_MoO_4_·2H_2_O (Sigma-Aldrich), Fe_2_(SO_4_)_3_ (Sigma-Aldrich), and Na_2_SO_4_ (Thermo Fisher Scientific) were used as potential methanogenic inhibitors, and sodium citrate (Na_3_C_6_H_5_O_7_·2H_2_O (Thermo Fisher Scientific)) was used as a potential methanogenic promoter.

### Mesocosm Setup

The experiment was conducted in 100 mL mesocosm bottles for Mesocosm A samples and 500 mL bottles for Mesocosm B samples. The difference in volume was due to the limited sample quantity for Mesocosm A samples. In total, 20 bottles of Mesocosm A and 20 bottles of Mesocosm B samples were prepared. Both Mesocosm A and Mesocosm B samples were incubated in the dark at room temperature, and methane (CH_4_) production in the headspace was monitored biweekly. Samples were also taken periodically from the mesocosms for chemical analyses and microbial community characterization. All the experimental bottles were flushed with nitrogen for 3 (100 mL bottles) or 20 min (500 mL bottles) after each sampling. The sampling frequency and schedule of events for the bottle test are shown in Figures 1 and S1.

**Figure 1:**
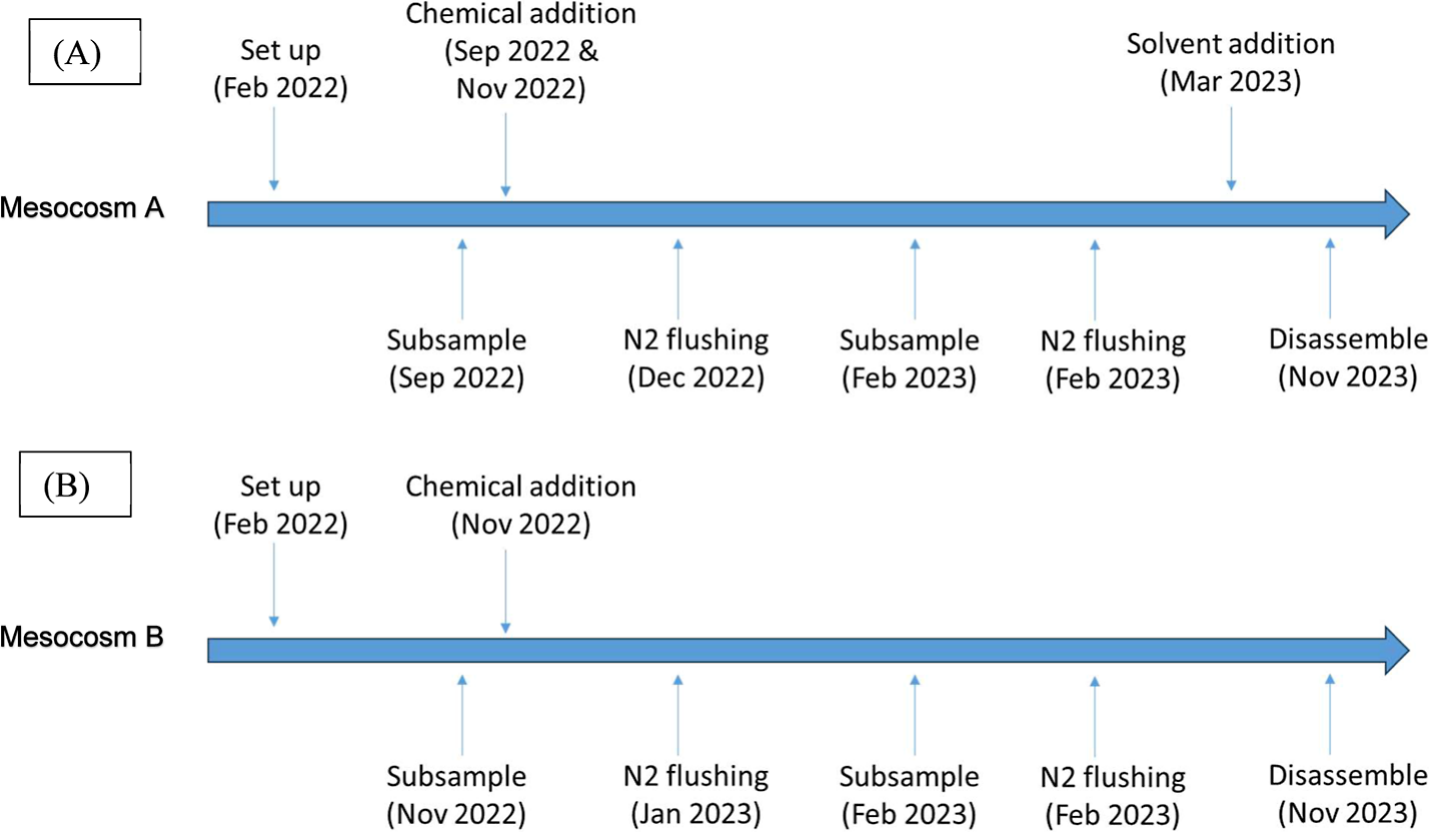
Sampling events during bottle test monitoring; (A) Mesocosm A bottles, (B) Mesocosm B bottles.

#### Mesocosm A Samples

The anaerobic mesocosms were prepared using 37.5 mL each of methanogenic medium and FFT in 100 mL serum bottles with a headspace of N_2_ as previously described.^12^ The methanogenic medium contained inorganic salts (NaCl, CaCl_2_, NH_4_Cl, MgCl_2_, (NH_4_)_6_Mo_7_O_24_·2H_2_O, ZnSO_4_, H_3_BO_3_, FeCl_2_, CoCl_2_, MnCl_2_, NiCl_2_, AlK­(SO_4_)_2_, NaHCO_3_, KH_2_PO_4_), vitamins (pyridoxine, thiamine, nicotinic acid, pantothenic acid, cyanocobalamin, *p*-aminobenzoic acid), sodium sulfide (reducing agent), and resazurin (redox indicator) as described previously.^13^ Each mesocosm also received 4 mL of mixed enrichment culture of methanogens, mainly as methanomicrobia, obtained from a previous study.^14^ The mesocosms were preincubated at room temperature in the dark for 2 weeks for microbial acclimation. Each treatment was prepared in duplicate. Two solvents, paraffinic and naphthenic, prepared in this study were used as sources of carbon to enhance microbial activity. The volume of the bottle was 100 mL containing 75 mL of materials. And each bottle received 0.12 g of solvent by weight.

#### Mesocosm B Samples

For Mesocosm B samples, the mesocosm was established using the tailings and process water from a previous bucket experiment. The bucket was prepared in 2020 using FFT and oil sands process water, both obtained from an industrial operator, with the addition of naphthenic solvent. It showed methane present in the headspace after one year of incubation at room temperature. In January 2022, the pail was opened in an atmospheric chamber, and the tailings and release water were weighed and subsampled. About 288.4 g of tailings and 111.7 g of released water were added to each 500 mL bottle, with the total weight of the materials of about 400 g. Similar to Mesocosm A samples, each treatment was prepared in duplicate, and both paraffinic solvent and naphthenic solvent were used as a source of carbon for the microbial community. And each bottle received 6.4 g of solvent by weight.

### Chemical Treatment

After reaching steady methane production from the bottles, the four chemicals at predetermined concentrations^2,10,11,15^ (shown in Table 1) were added into the duplicate bottles using a sterile plastic syringe. Efforts were made to add the chemicals in multiple portions at different depths or at different locations inside the bottles to ensure the chemicals can be evenly distributed throughout the tailing samples. After chemical addition, these bottles were homogenized by a shaker for 30 min. Duplicate baseline controls (unamended mesocosms) were also prepared to monitor CH_4_ production without the addition of any chemicals as a methane suppressant/stimulant. The number of bottle samples under different conditions is given in Table S1.

**Table 1:** Chemical Addition to the Bottles

chemicals	molecular weight (g/mol)	stock concentration (g/L)	required sulfate or molybdate or citrate concentration in tailings (mM)^2,10,11,15^	volume (mL) delivered to Mesocosm A samples in Sep 2022	volume (mL) delivered to Mesocosm A samples in Nov 2022	volume (mL) delivered to Mesocosm B samples in Nov 2022
Na_2_MoO_4_·2H_2_O	241.95	763	20	0.38	0.44	2.11
Fe_2_(SO_4_)_3_	399.88	256	52	1.62	1.90	9.02
Na_3_C_6_H_5_O_7_·2H_2_O	294.10	770	20	0.46	0.53	2.54
Na_2_SO_4_	142.04	281	52	1.58	1.83	8.75

### Headspace Methane Analysis

During the bottle tests, positive headspace pressure was mostly maintained due to methane production. Methane was measured by taking out 100 μL headspace with a syringe and injecting it into a gas chromatograph for analysis. An Agilent 7890A+ gas chromatograph equipped with a flame ionization detector (GC-FID) and an Agilent Technologies HP-5MS column (30 m × 0.25 mm × 0.25 μm) was employed. The inlet temperature was set to 80 °C at a split ratio of 50:1. The oven temperature was held constant at 80 °C, and the total run time was 1.4 min.

### Tailings Analysis

#### Dean–Stark Analysis

The concentrations of bitumen, mineral solids, and water content of a tailing sample were quantitatively determined by Dean–Stark analysis,^16^ where bitumen was extracted from the sample by heating toluene using the modified Soxhlet extractor apparatus. During the extraction process, the solids remained in the thimble; the water was collected in the water trap, and the bitumen was collected in the boiling flask, together with the toluene. At the end of the extraction, the weights of bitumen, solids, and water in the sample were acquired by using an analytical balance. In this study, Dean–Stark analysis was performed on FFT prior to bottle assembly.

#### Capillary Suction Time

Capillary suction time (CST) was measured using a multipurpose capillary suction time apparatus from Triton Electronics (Type 319 multi-CST). The multiradi test head has 5 probes, and each sample was analyzed in triplicate. The tailings material was manually mixed and then pipetted into the CST tube (5 cm tubes were used) until it was completely full.

The CST apparatus was set up using Whatman filter paper cut into quarters and 5 cm tubes. The rate at which water permeated through the filter paper was measured by the 2 electrodes of the CST apparatus. The time required for water to travel between the two electrodes defined the capillary suction time, which was measured in seconds.

### Water Chemistry Analysis

Depending on the sample volume, selected water chemistry analysis was performed using free surface water (i.e., decant water) or pore water samples from FFT and mesocosms. Pore water samples were collected after centrifuging the tailings/slurry at 25,000 rpm for 20 min.

Solution alkalinity, pH, and electrical conductivity were measured with a Man-Tech Associates PC-Titrate instrument equipped with a TitraSip module, which was calibrated using a Waters ERA P272-506 standard.

The concentrations of dissolved calcium, sodium, potassium, magnesium, sulfur and trace metals were measured using a Varian Vista-Pro 725 radial simultaneous inductively coupled plasma optical emission spectrometer (ICP-OES) equipped with a SPS3 autosampler. The working standards of each analyte were prepared by using certified standard stock solutions.

The concentrations of chloride, nitrate, fluoride, and sulfate were measured with a Thermo-Fisher ICS 3000 ion chromatography (IC) system. The working standards of each analyte were prepared by using certified standards diluted with deionized water. A commercial standard containing 40 ppm sulfate and 10 ppm each chloride, nitrate, and fluoride was used as a quality control standard.

### Microbial Analysis

Microbial analysis was performed on the collected FFT and mesocosm samples before methane production and during methane production and chemical treatment (in early 2022, late 2022, February 2023, and November 2023) to establish the microbial community profile. For DNA extraction, tailings samples were centrifuged for 5 min at 4500 *x* *g*, and following centrifugation, the supernatant was filtered through 0.22 μM disk filters (Millipore Express, filter diameter 47 mm, hydrophilic poly­(ether sulfone) membranes). DNA was extracted from filters using a modification of the hexadecyltrimethylammonium bromide method as described in Tremblay et al.^17^ The extracted DNA was sequenced using commercial kits (DNeasy PowerWater Kit). The primers of both 515F-Y (5-GTGYCAGCMGCCGCGGTAA-3) and 926R (5-CCGYCAATTYMTTTRAGTTT-3), as well as 515F (5-GTGCCAGCMGCCGCGGTAA-3) and 806R (5-GGACTACHVGGGTWTCTAAT-3), were used to target Archaea and Bacteria.^18^ Biomolecular analysis was performed on extracted samples to evaluate the microbial community diversity and relative abundance (RA) of the various taxa present in samples via high-throughput 16S rRNA gene sequencing. The sequencing and quantitative assay followed previously reported protocols.^19^ Raw data obtained from the sequencing were processed using the Illumina MiSeq platform (Illumina Inc., San Diego, CA),^20^ and detailed procedures are available elsewhere.^21^ Relationships between microbial taxa and treatments were established based on the relative abundances of the microbial community obtained from 16S rRNA gene sequencing and visualized using XLSTAT software.

### Subsample for Various Analyses

50 mL FFT samples used to setup mesocosms were collected in early 2022 before assembling the bottles for chemical and microbial analyses. They are considered “before assembly” or “before solvent” samples in this study. After bottle assembly and solvent addition, 5 mL mesocosm samples were collected from each bottle in late 2022 before chemical treatment. The Mesocosm A samples with the same solvent type were combined and centrifuged to pool the pore water for chemical and microbial analyses. Similar procedures were performed on Mesocosm B samples. These samples were labeled as “combined sample PAR” and “combined sample NA” in this study, with PAR representing paraffinic and NA representing naphthenic. During chemical treatment in Feb 2023, 2 mL mesocosm samples in each bottle were collected for microbial analysis; 10 mL mesocosm A samples and 15 mL Mesocosm B samples in each bottle were subsampled for chemical analysis, respectively. At the end of the bottle test, all of the samples were consumed for various tests. Thus, during chemical treatment, given that there was only a minimal loss of samples compared to the total volume of the materials in each bottle, this reduction in volume should not affect the methane treatment process. In addition, during the bottle test, after each subsample, N_2_ flushing was performed to reset anaerobic conditions and the accumulated methane to zero.

## Results and Discussion

### Bottle Setup

Compared to Mesocosm A samples, the composition of Mesocosm B samples had more tailings but less water (∼72% vs 50%). Tailings provide indigenous methanogens. Since mesocosm A received 4 mL of mixed enrichment culture of methanogens, less amount of indigenous methanogens would be needed for the mesocosm preparation. In addition, Mesocosm B samples contained approximately 1.6 wt % of solvent primarily in the hope of boosting the methane production, while Mesocosm A samples contained approximately 0.16 wt % of solvent to simulate the FFT in the real-world application. It is reported that FFT are a colloidal slurry composed of >90 wt % slightly alkaline water, 8–12 wt % solids, 5 wt % bitumen, and <0.5 wt % unrecovered solvent and are retained in tailings ponds pending reclamation,^22^ whereas froth treatment tailings, generated during the bitumen froth separation process, are typically composed of 76.5 wt % water, 17 wt % mineral solid particles, 4.5 wt % bitumen, and up to 2 wt % diluent.^23^ Therefore, Mesocosm A samples are closely representative of FFT and Mesocosm B samples are similar to froth treatment tailings. As oil sands tailings ponds receive both streams of tailings, the large difference in the amount of solvent in our mesocosm samples will provide us some insight into the treatment effects given the heterogeneous environment in the oil sands tailings ponds.

Compositions of FFT used for mesocosm preparation were measured by Dean–Stark analysis and are listed in Table 2. As the sources of the FFT were different between Mesocosm B samples and Mesocosm A samples, it is expected that the pore water chemistry of tailings would also be different between the two mesocosms. In this study, the water chemistry of the pore water of both FFTs and the surface water of the Mesocosm B FFT is shown in Table 3 and Figure 2. It appears that the total dissolved solids (TDS) level of the Mesocosm A FFT pore water was higher than that of the Mesocosm B FFT pore water. In addition, as shown in Figure 2, the trace metal contents of the pore water were similar between the two types of FFT, except for selenium and zirconium, which were present at higher concentrations in the Mesocosm A FFT.

**Table 2:** Composition of Fluid Fine Tailings (FFT) before the Bottle Assembly

samples	% bitumen	% water	% solids	mass balance (% bitumen + % water + % solids) (%)
FFT for Mesocosm A	2.97	53.35	43.48	100.0
FFT for Mesocosm B	3.96	59.08	36.95	99.8

**Table 3:** Major Water Chemistry for FFT and Water Samples Used for Mesocosm Bottle Assembly

samples	conductivity (μS/cm)	HCO_3_^–^ (mg/L)	CO_3_^2–^ (mg/L)	OH^–^ (mg/L)	pH	TDS, calculated (mg/L)	Cl^–^ (mg/L)	F^–^ (mg/L)	nitrate (as N) (mg/L)	SO_4_^2–^ (mg/L)	Ca^2+^ (mg/L)	K^+^ (mg/L)	Na^+^ (mg/L)	Mg^2+^ (mg/L)
Mesocosm A FFT pore water	3850	1470	24.2	<1.0	8.5	2420	608	3.6	<0.10	6.7	21.9	19.1	985	14.7
Mesocosm A FFT-media-inoculum^a^					8.4		1099.2	9.6	<25	208.8	30.6	87.9	1667.7	23.9
Mesocosm B FFT pore water	1370	650	3.7	<1.0	8.3	838	120	2.1	0.04	5.5	20.2	18	324	14.5
Mesocosm B FFT decant water	1380	592	27.5	<1.0	8.8	856	126	1.9	0.05	3.9	9.9	16.3	341	16.8

a Some water chemistry data unavailable due to limited sample quantity; this sample was collected following centrifugation of a mixture of FFT, media, and inoculum corresponding to the bottle assembly composition.

**Figure 2:**
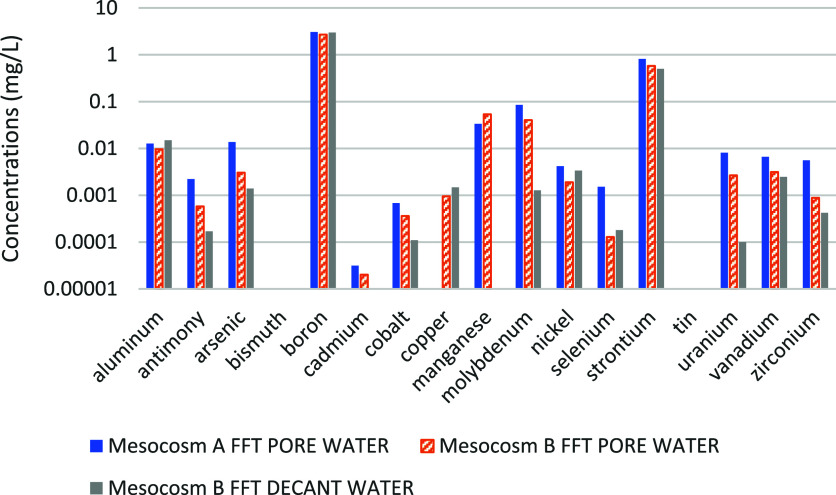
Trace metal analysis for FFT and water samples used for mesocosm bottle assembly.

### Headspace Methane Monitoring

The temporal evolution of methane production in the headspace of the Mesocosm A bottles and Mesocosm B bottles is shown in Figures S2 and S3. In this study, we named this initial methane production phase as phase 1. Results indicate that the methane content in the headspace of the bottles reached the steady rate of production at about 2 months after the initial bottle setup for Mesocosm A tailing samples, whereas the methane production process for Mesocosm B tailings samples was a bit slower, which took approximately 6 months to reach the same level. The reason for the difference in the rate of methane production between the two mesocosms was likely the different quantities of solvents added into the bottles during the initial setup. Mesocosm A received 0.16% by weight of solvents, whereas Mesocosm B received 1.6% by weight of solvents. The higher quantity of solvents in the Mesocosm B bottles likely created a temporary inhibitory effect on the methanogens in the tailings, therefore inhibiting methane production.^24^ In addition, Mesocosm A received a mix culture of methanogens, while Mesocosm B only received the indigenous bacteria from the tailings. This could also contribute to the difference in methane production observed in this study.

With regard to the effect of solvents on CH_4_ production, it appears that Mesocosm A samples fed with naphthenic solvent produced a higher quantity of CH_4_ than the ones that received paraffinic solvents. We speculate that the Mesocosm A tailings materials were obtained from a naphthenic oil sands producer; therefore, the indigenous bacteria in the Mesocosm A tailings were able to adapt to the naphthenic solvent environment more easily and generate CH_4_ at a faster rate. On the other hand, the Mesocosm B samples that received paraffinic solvent produced more CH_4_ than the samples that received naphthenic solvents. The reason for this may be due to the long-lasting toxicity effects of the naphthenic solvents compared to the paraffinic solvents, which further slows the methane production from the naphthenic tailings. But both solvents have stimulated methane production in the mesocosms in our study.

It is reported in the literature that mesocosms amended with paraffinic components had a longer lag time for methane production than the ones amended with naphthenic components.^22,25^ In addition, these previous studies highlight the differences in CH_4_ emissions associated with the type of diluent used. Nevertheless, CH_4_ were produced from tailings spiked with either 0.2 wt % naphthenic or 0.1 wt % paraffinic diluent.^22,26^ Since higher diluent/bitumen ratios were required for the paraffinic froth treatment process,^27^ one would expect more methane to be emitted from the paraffinic tailings ponds. However, as mentioned earlier, this is not the case based on the current industrial reporting.^7^ Therefore, other factors may contribute to the difference in emissions, such as the amount of solvent in the pond, sampling location, operational practices, tailings management strategies, and site-specific conditions. This is similar to what we observed in our mesocosm studies. Without knowing the details, it is difficult to extrapolate the quantitative emissions from different mesocosms to tailings ponds. As a result, the reasons behind the different amounts of fugitive emissions from tailings ponds under different froth treatment processes should be further investigated.

As shown in Figures 1, S2, and S3, the chemical addition was made at the end of phase 1. In particular, four different treatment chemicals (Na_3_C_6_H_5_O_7_·2H_2_O, Na_2_MoO_4_·2H_2_O, Fe_2_(SO_4_)_3_, and Na_2_SO_4_) at predetermined concentrations were selected based on the literature review to inhibit or promote the methane gas production.^2,10,11,15^ However, these four treatment chemicals have never been applied to oil sands tailings samples. It is to be noted that Mesocosm A bottles received 2 doses of chemical additions in series, and Mesocosm B bottles only received 1 dose of chemical addition (Figure 1). In fact, the Mesocosm A samples produced more CH_4_ in phase 1; therefore, the first chemical addition was made earlier than in Mesocosm B bottles, about 6 months after the initial bottle setup. However, after chemical addition, the methane production in the headspace of these bottles still continuously increased, after an initial sharp drop, likely due to the interruption of the anaerobic condition during the chemical addition process. Hence, a second chemical addition to the Mesocosm A bottles was made 2 months after the first chemical addition, which was at the same time as the chemical addition time point of the Mesocosm B bottles.

After chemical addition at the end of phase 1, N_2_ flushing was performed to reset the anaerobic conditions and the accumulated methane to zero. This is when phase 2 of the methane production monitoring started. As shown in Figure 3, for Mesocosm A bottles, the difference in the headspace methane production was clearly observed within the first 2 months of phase 2 monitoring, with respect to different chemical treatments. Mesocosms that received sodium citrate (Na_3_C_6_H_5_O_7_·2H_2_O) have higher methane production in the headspace for both the Mesocosm A naphthenic bottles and Mesocosm A paraffinic bottles than the other chemical treatments. The other three chemicals (Na_2_MoO_4_·2H_2_O, Fe_2_(SO_4_)_3_, and Na_2_SO_4_) seem to decrease the methane production in the headspace of the bottles compared to the control experiments without chemical treatment, particularly in the paraffinic bottles. However, after the first 2 months of monitoring in phase 2, the methane production decreased dramatically in Mesocosm A samples, including the control samples, suggesting solvents fueling this activity were completely consumed. Accordingly, at the end of phase 2 monitoring, solvents were added to the Mesocosm A bottles at a concentration of 0.16% by weight to once again provide sufficient nutrients for the microbial community in the mesocosms in order to continue the long-term bottle test monitoring.

**Figure 3:**
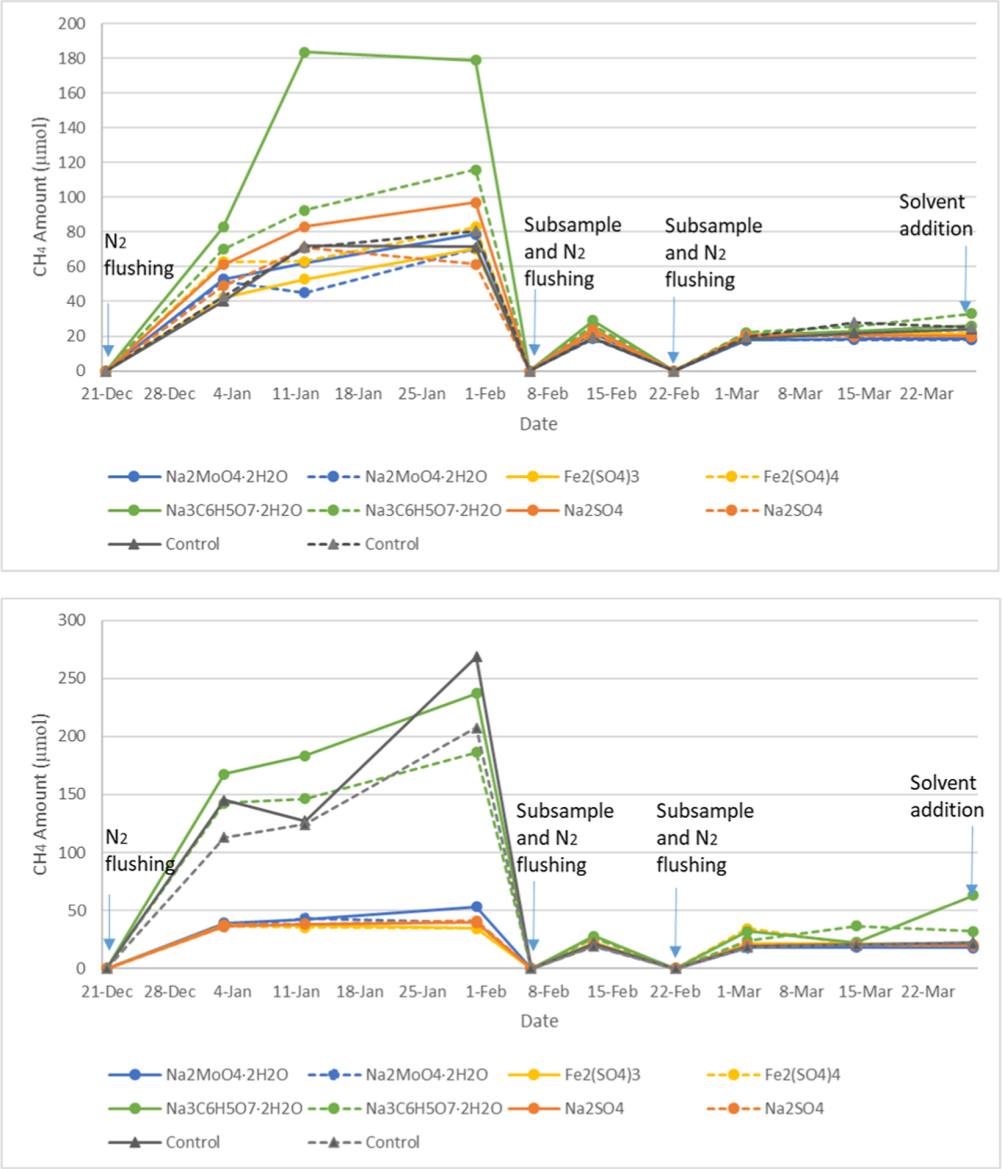
Temporal evolution of methane production in the headspace of the Mesocosm A naphthenic bottles (top chart) and paraffinic bottles (bottom chart) in phase 2; treatments are shown as colored dotted lines, with duplicates represented by solid and dashed lines of the same color.

After this solvent addition, phase 3 monitoring begins for Mesocosm A bottles, and the results are shown in Figure 4. When compared to the control experiments, it appears that the three chemicals (Na_2_MoO_4_·2H_2_O, Fe_2_(SO_4_)_3_, and Na_2_SO_4_) inhibited methane production. However, for sodium citrate (Na_3_C_6_H_5_O_7_·2H_2_O), although it increased the concentration of methane in the headspace of the bottles compared to the other three chemicals, the amount of methane produced is similar to that in the control experiments. Given that sodium citrate (Na_3_C_6_H_5_O_7_·2H_2_O) led to increased methane production in phase 2, it can be inferred that this compound promotes methanogenesis in the context of this study. With this in mind, one could have expected methane accumulation in Mesocosm A with sodium citrate exceeding that of the control bottles. Therefore, the similarity in methane production observed between Mesocosm A treated with sodium citrate and the control is likely due to the fact that sodium citrate, the bacterial food source in Mesocosm A, was consumed in phase 2 since these bottles in phase 3 received no additional chemical treatment.

**Figure 4:**
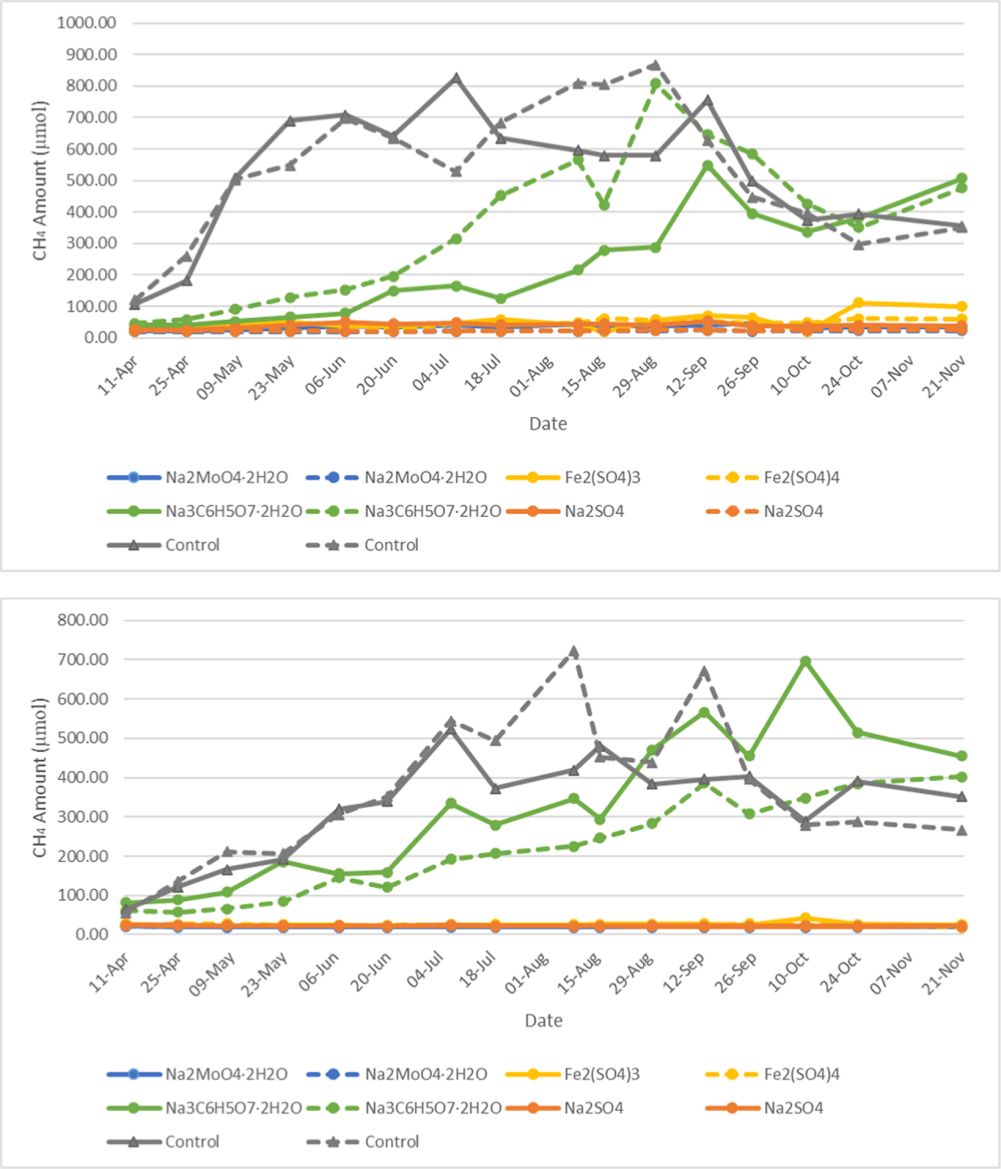
Temporal evolution of methane production in the headspace of the Mesocosm A naphthenic bottles (top chart) and paraffinic bottles (bottom chart) in phase 3; treatments are shown as colored dotted lines, with duplicates represented by solid and dashed lines of the same color.

In addition, methane production in phase 3 was higher for the Mesocosm A naphthenic control bottles than for the Mesocosm A paraffinic control bottles. The observed delayed methane emission in the paraffinic solvent control samples, compared to the naphthenic solvent control samples, is consistent with findings observed in phase 1 and reported in the literature.^22,25^ At the same amount of solvent addition for both naphthenic and paraffinic bottles, bottles containing naphthenic solvents are expected to receive a higher carbon input due to the heavier and more complex molecular structures, compared to paraffinic solvents; therefore, for Mesocosm A samples, naphthenic bottles have higher amounts of methane produced than paraffinic bottles.

For Mesocosm B samples, due to the larger quantity of solvents added during the initial bottle setup, unlike Mesocosm A samples, there was no need for the additional solvent amendment. As shown in both phases 2 and 3 (shown in Figures 5 and 6), methane production was high in the mesocosms that received sodium citrate treatment. Phase 2 and phase 3 were supposed to be a continuous process. But due to the subsampling, the baseline was reset at the end of phase 2. For both sets of Mesocosm B naphthenic bottles and Mesocosm B paraffinic bottles, the increase of methane production by sodium citrate lasted for 5–6 months (from February to September 2023 in phase 3, Figure 6). This indicates that the dosage of sodium citrate was consumed within this time frame. Unlike Mesocosm A bottles, the consumption of sodium citrate of Mesocosm B bottles was at a much slower rate, probably because of the initial inoculum enrichment culture added in the Mesocosm A bottles, where the microbial community quickly adapted to the new environment and consumed the readily available labile hydrocarbons; in contrast, Mesocosm B contained only indigenous bacteria from the tailings, and the resulting environmental changes led to a slower methane production process. In addition, the other 3 chemicals (Na_2_MoO_4_·2H_2_O, Fe_2_(SO_4_)_3_, and Na_2_SO_4_) successfully inhibited the methane production during the entire phase 3. The control samples of naphthenic amended Mesocosm B showed an increase in methane production during the late stage of phase 3 monitoring. However, there was no elevated methane production observed for the control samples of paraffinic amended Mesocosm B. This confirms the long lag time for methane production in paraffinic amended bottles and indicate the microbes in the FFT from a naphthenic producer may have difficulties of growing in the new paraffinic solvent environment.

**Figure 5:**
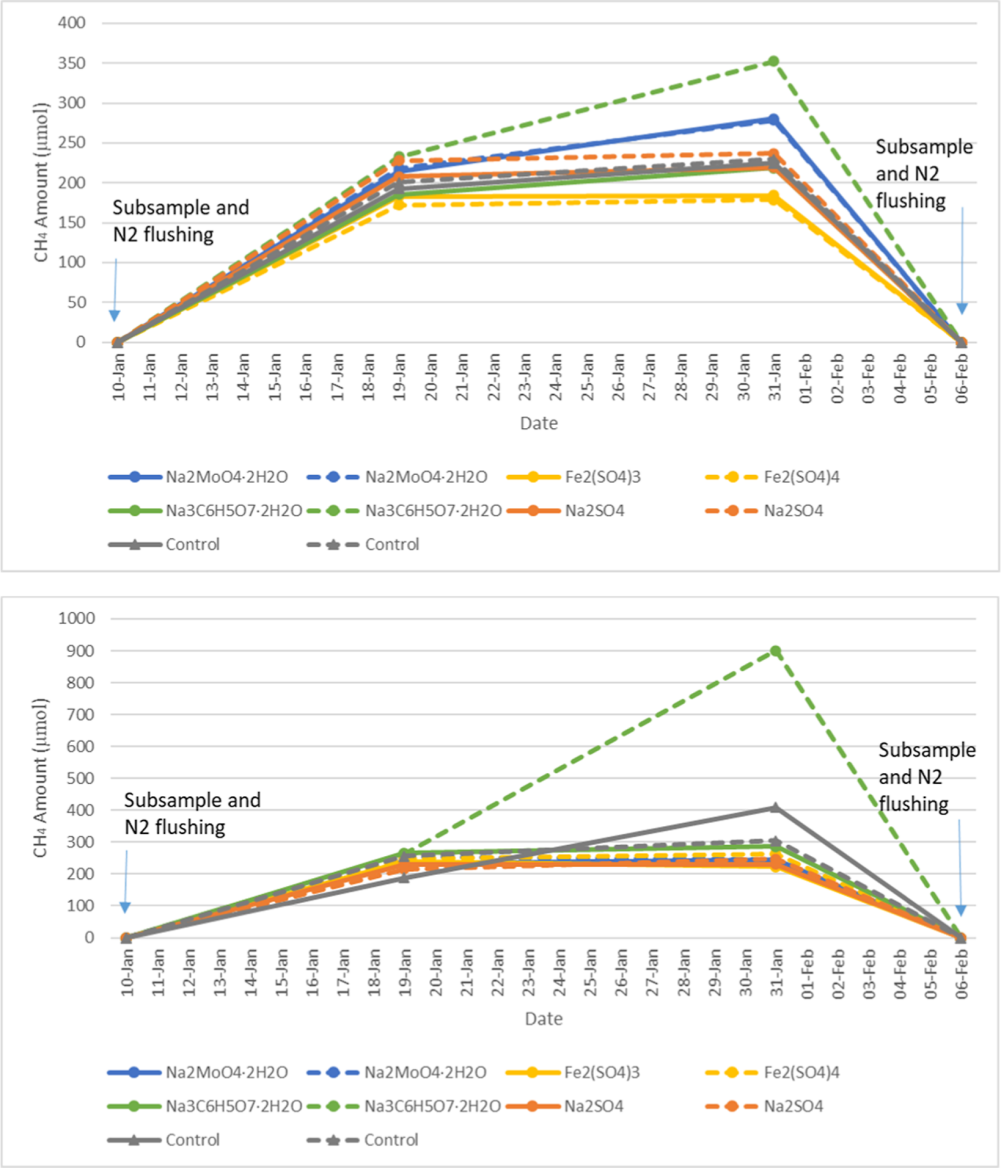
Temporal evolution of methane production in the headspace of the Mesocosm B naphthenic bottles (top) and paraffinic bottles (bottom) in phase 2; treatments are shown as colored dotted lines, with duplicates represented by solid and dashed lines of the same color.

**Figure 6:**
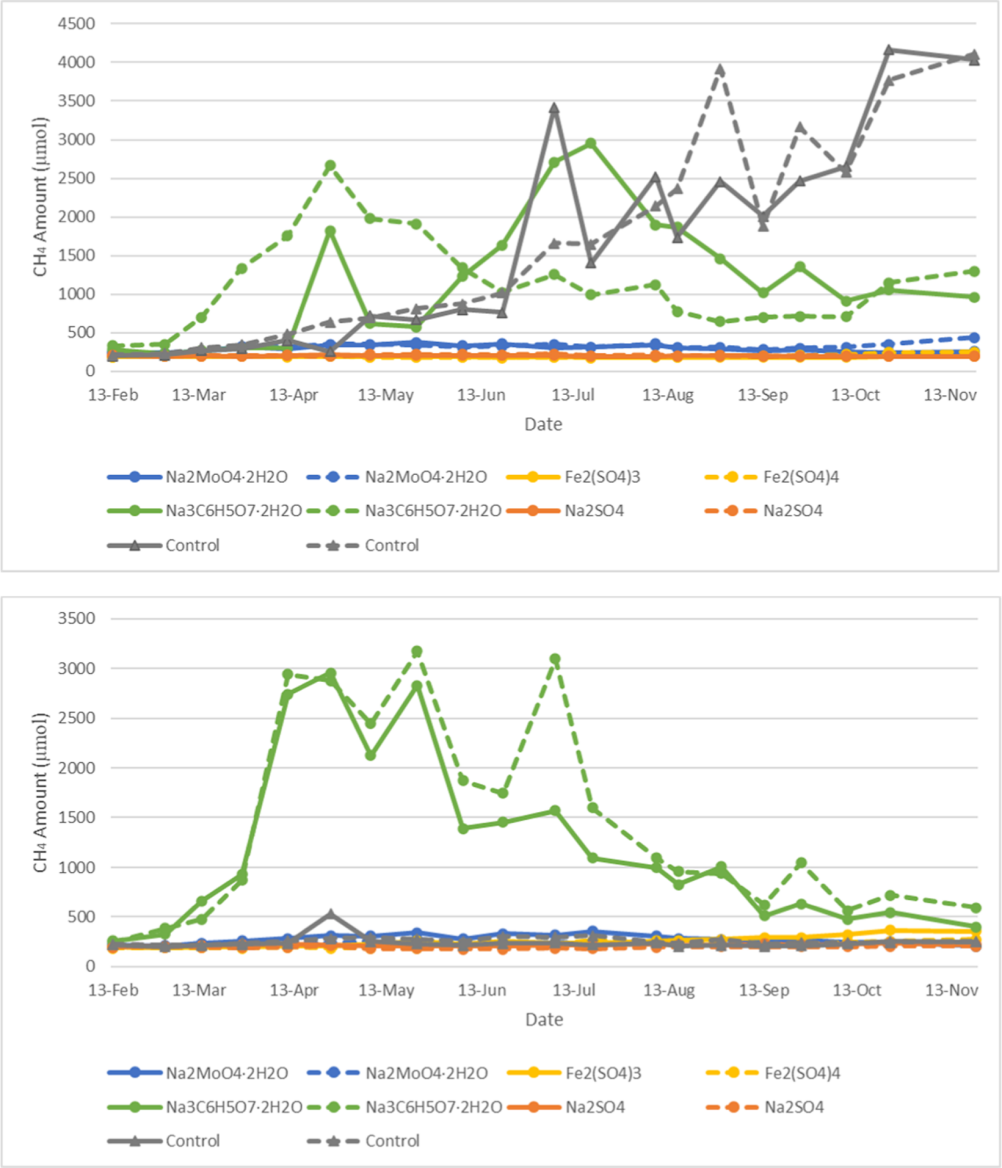
Temporal evolution of methane production in the headspace of the Mesocosm B naphthenic bottles (top) and paraffinic bottles (bottom) in phase 3; treatments are shown as colored dotted lines, with duplicates represented by solid and dashed lines of the same color.

In addition, for naphthenic amended Mesocosm B bottles, the methane production of sodium citrate-treated samples peaked earlier than their respective control samples (as shown in Figure 6). At the end of the monitoring period, the peak reached its lowest point, which may indicate that the solvent, sodium citrate, or both have been completely consumed in the experiment. However, the amount of methane in the control experiments of the naphthenic amended bottles seems to reach its peak value at the end of phase 3. Therefore, it is difficult to determine the time frame of the entire methane production process for these control samples owing to the early termination of experiments. Nevertheless, when comparing the size and area of the peaks between the sodium citrate samples and the control samples, it appears that there was still enough solvent in the sodium citrate-treated samples after the lowest point of the peak. Therefore, it is highly probable that sodium citrate rather than solvent was completely consumed in the process. For Mesocosm B paraffinic amended bottles, the control samples barely had any methane production, which was similar to the rest of the chemical inhibited samples. The sodium citrate sample was the only one that peaked the methane emission during the phase 3 monitoring period. It appears that sodium citrate enhanced the ability of indigenous bacteria to more effectively and rapidly tolerate the toxic effects associated with a high solvent dosage. Thus, it is expected that with a longer monitoring period methane production will be initiated in the control samples once the lag time has passed and the toxicity effects of the high dose solvent are further tolerated.

### Water Chemistry Profile during Chemical Treatment

The water chemistry of the bottle samples was compared. As shown in Figure 7, an increase in the concentrations of Ca and Mg was observed in the pore water shortly after Fe_2_(SO_4_)_3_ addition in February 2023 (phase 2). Oil sands tailings contain clay minerals. It is known that the clay surfaces retain ions, with Ca, Mg, and Na all being common.^28^ Thus, when a larger charge ion, such as Fe^3+^, enters the solution, it can displace these retained ions from the surface, resulting in elevated concentrations of Ca and Mg in solution. The cation exchange mechanism could be due to the lyotropic series; the relative strength of ion adsorption onto charged soil colloids is stronger for the polyvalent ions.^29^

**Figure 7:**
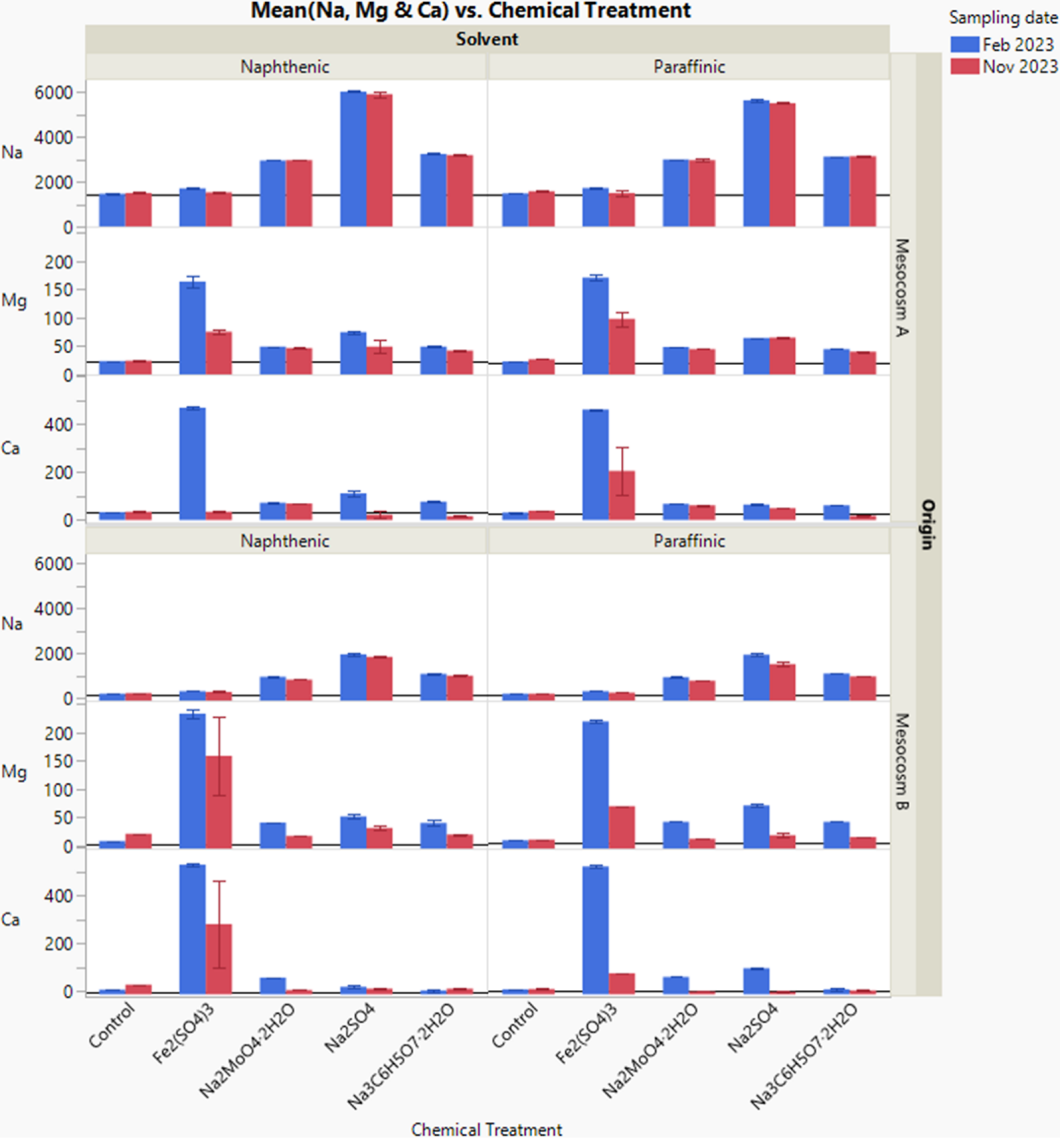
Comparing the concentrations (in mg/L) of Na, Mg, Ca in the pore water of different bottle samples following chemical treatments; control: without chemical treatment; error bar represents the minimum and maximum of the data. Reference lines in each graph demonstrate the concentrations of the elements in “before treatment” samples.

In addition, Figure 8 shows the pH of the pore water decreased dramatically from 8 to about 4 following Fe_2_(SO_4_)_3_ addition in Feb 2023. This change in pH was not observed after the addition of the other chemicals. The pH drop was primarily due to Fe^3+^ hydrolysis with the delayed effect of microbial processes such as iron reduction and sulfate reduction.

**Figure 8:**
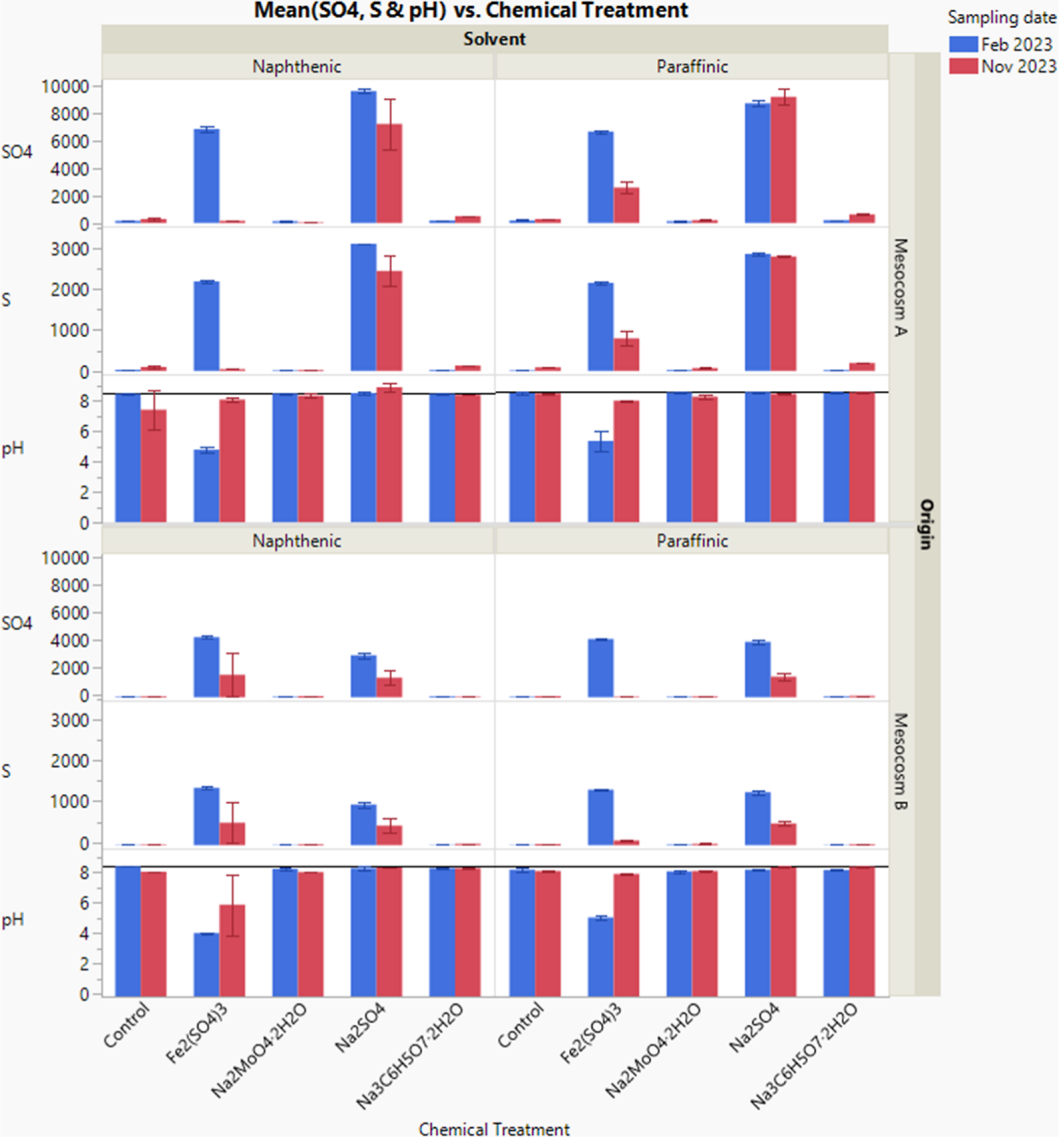
Comparing the concentrations of sulfate (mg/L), sulfur (mg/L), and pH in the pore water of different samples following chemical treatments; control: without chemical treatment; error bar represents the minimum and maximum of the data. Reference lines in the graphs demonstrate the pH in “before treatment” samples.

At the end of the sample monitoring period in November 2023 (phase 3), the concentrations of Ca and Mg elements in Fe_2_(SO_4_)_3_ addition samples were largely reduced compared to that in February 2023 samples. In addition, sulfate and sulfur concentrations in the majority of Fe_2_(SO_4_)_3_ and Na_2_SO_4_ addition samples were reduced compared to that in February 2023 samples, which indicates the consumption of these chemicals during the methane inhibition process. And pH of the pore water increased with the consumption of the Fe_2_(SO_4_)_3_ chemicals, likely due to the microbial reactions induced by iron-reducing bacteria and the sulfate-reducing bacteria. It appears that paraffinic Mesocosm B samples and naphthenic Mesocosm A samples have completely consumed Fe_2_(SO_4_)_3_ by or before November 2023 (Figure 8).

Furthermore, as shown in Figure 1, the Mesocosm A samples received equal amounts of chemical addition in both September 2022 and November 2022, while the Mesocosm B samples received only one chemical addition in November 2022. Therefore, based on mass balance calculation, the total amounts of SO_4_^2–^ and S added to the Mesocosm A samples through both Fe_2_(SO_4_)_3_ addition and Na_2_SO_4_ addition were supposed to be 9271 and 3090 mg/L, respectively; the total amounts of SO_4_^2–^ and S added to the Mesocosm B samples were supposed to be 4992 and 1664 mg/L, respectively.

As shown in Figure 8, the measured amounts of SO_4_^2–^ and S in the naphthenic Mesocosm A samples treated by Fe_2_(SO_4_)_3_ were 6821 and 2193 mg/L in February 2023, respectively, whereas the concentrations of SO_4_^2–^ and S were 9589 and 3113 mg/L, respectively, in the Mesocosm A samples treated by Na_2_SO_4_ measured at the same time. Since the initial amounts of SO_4_^2–^ and sulfur introduced from both Fe_2_(SO_4_)_3_ and Na_2_SO_4_ additions were the same, the results indicate that, for naphthenic Mesocosm A samples, Fe_2_(SO_4_)_3_ got consumed at a faster rate than Na_2_SO_4_. Similarly, for paraffinic mesocosms, the measured amounts of SO_4_^2–^ and S in the Mesocosm A samples treated by Fe_2_(SO_4_)_3_ were 6632 and 2155 mg/L in February 2023, respectively, whereas the concentrations of SO_4_^2–^ and S in Na_2_SO_4_ treated samples were 8687 and 2861 mg/L, respectively. This confirms that Fe_2_(SO_4_)_3_ was consumed at a faster rate than Na_2_SO_4_ independent of the solvent used and paraffinic bottle samples consumed sulfate at a similar rate compared to naphthenic bottle samples in phase 2. However, in November 2023, although Fe_2_(SO_4_)_3_ continued to be consumed at a faster rate than Na_2_SO_4_, since Fe_2_(SO_4_)_3_ was nearly depleted in naphthenic Mesocosm A samples, it indicates naphthenic mesocosm samples consumed the chemicals at a faster rate than the paraffinic samples. This may be due to the long monitoring period, which demonstrates that the solvents may play a role in the chemical treatment process. In addition, the water chemistry data correspond well to the methane emission data, where naphthenic bottles have higher amounts of methane produced than paraffinic bottles, thus consuming chemicals at a faster rate.

For Mesocosm B samples, different water chemistry characteristics have been observed. In February 2023, the amounts of SO_4_^2–^ and S in Fe_2_(SO_4_)_3_ addition samples were equal to or slightly higher than those in Na_2_SO_4_ addition samples, indicating the same consumption rate for both chemicals. By November 2023, the same trend holds, except that paraffinic bottle samples have consumed nearly all of the Fe_2_(SO_4_)_3_, which was opposite to the Mesocosm A samples. Considering that Mesocosm B has a slower microbial activity due to the toxic effects induced by the high concentration of solvent, these results indicate that a different microbial community profile could be available in the Mesocosm B samples compared to the Mesocosm A samples, which may be attributed to the different consumption rate of SO_4_^2–^.

The faster consumption rate of Fe_2_(SO_4_)_3_ may indicate the stronger methane inhibition in the bottle samples compared to the other chemical treatments. This is likely due to the synergy effect of the sulfate reducing bacteria and iron reducing bacteria. In addition, in all of the bottle samples, S concentration was always at about 1/3 of the concentration of SO_4_^2–^, indicating minimal amount of H_2_S if any has been produced in the system. Although direct H_2_S measurement was not performed in this study, given that the pH values in most samples were around 8, the sulfur produced by sulfate-reducing bacteria in these samples would likely form iron sulfide (FeS), cosettling with the tailings and thus reducing potential environmental risks.

### Tailings Analysis—Capillary Suction Time Analysis

Oil sands tailings consist mostly of fine clay particles suspended in a dilute aqueous solution of sodium hydroxide and surfactants formed during the extraction process.^30^ Although the solids settle over a period of weeks or months, they do not settle completely. A sludge is formed consisting of around 30% solids, which does not settle further over a reasonable timespan. The suspended clay particles are held apart from each other by strong repulsive electrostatic forces. In addition, because the particles are so fine (less than 2 μm), water does not easily flow away from the particles, even under capillary action.

Capillary suction time (CST) is a simple and precise measure of the rate at which water is released from a sludge matrix. This measure of sludge dewaterability is used to optimize the performance and operation of sludge dewatering processes, including the calculation of optimal chemical addition to aid separation.^31^ Sludges that release water quickly exhibit a low CST, whereas those that release water slowly have a high CST. The test is used primarily to indicate filter cake permeability, but data from the test have been used to study the consolidation and filterability properties of oil sands tailings.

As shown in Figure 9, a low CST was observed for tailing samples after the Fe_2_(SO_4_)_3_ treatment. Compared to the control samples, the CST was decreased by more than 50% for both Mesocosm B and Mesocosm A samples under the condition of either naphthenic solvent or paraffinic solvent. However, the decrease in CST was not observed in the other chemical-treated samples. The result indicates good dewaterability for the Fe_2_(SO_4_)_3_-treated tailing samples. Historically, many methods have been tested with a view to finding a way of either causing the fluid fine tailing in the tailings ponds to settle into solid and liquid phases or increasing the filterability to an acceptable level so that the liquid can be removed by filtration.^14^ The data from this study suggest that Fe_2_(SO_4_)_3_ could be used to achieve both methane inhibition and tailings consolidation.

**Figure 9:**
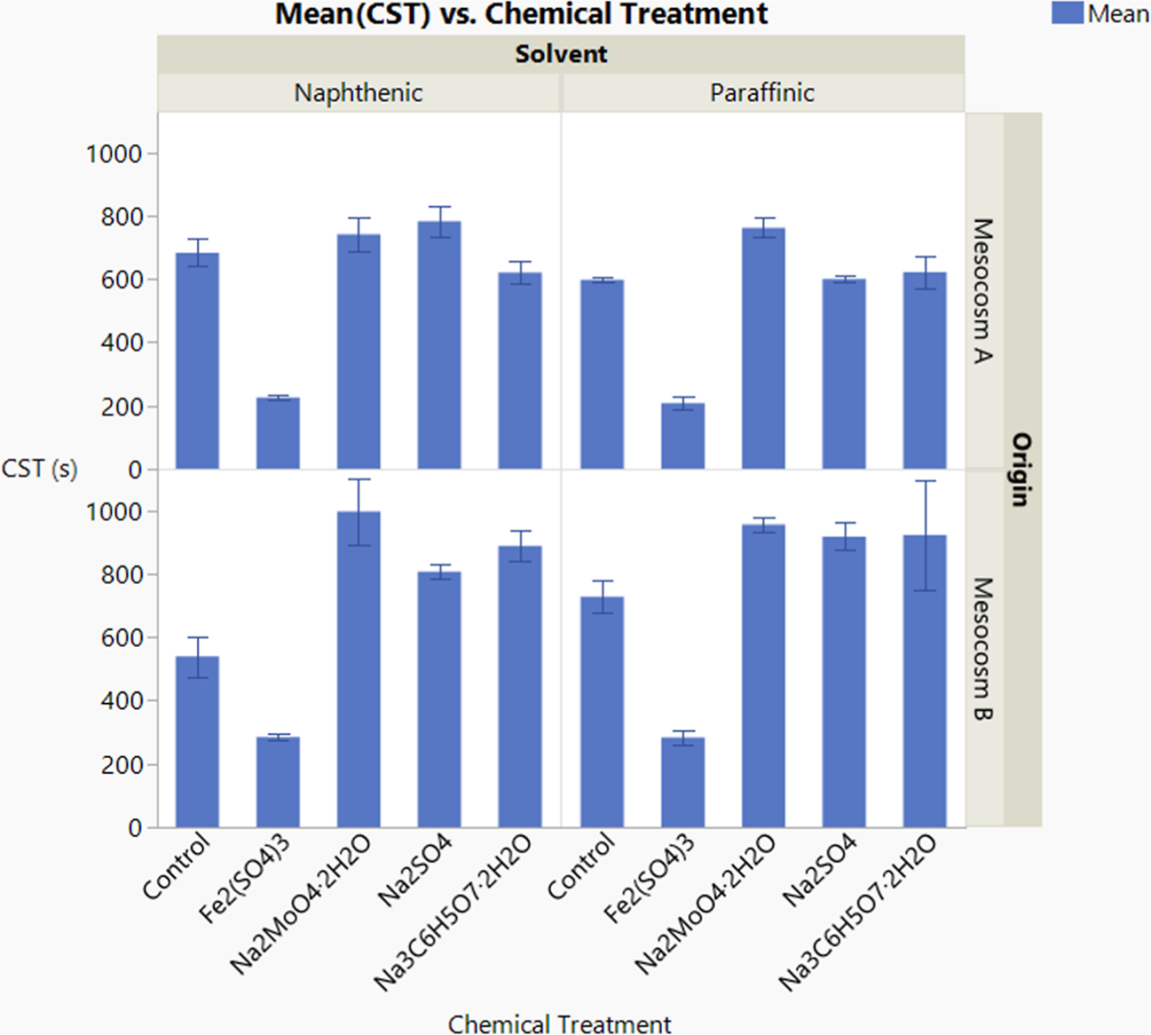
Capillary suction time (CST) analysis for the tailing samples after different chemical treatments (sampled in Nov 2023, phase 3). Error bar represents the minimum and maximum of the data.

### Microbial Response to Solvents and Chemical Treatments

Analysis of the microbial community composition in the tailings showed that bacteria and archaea remained dominant (Figure S4 and Table S2) across all samples, including pretreatment, at the end of phase 2 (February 2023) and at the end of phase 3 (November 2023). The abundance of bacteria was considerably higher than the archaea across all samples. At the end of phase 2 (February 2023; Figure S4), archaea and bacteria comprised 9.09 ± 2.51% and 83.79 ± 3.66% of the taxa in the Mesocosm B mesocosm samples (Table S2), respectively. By the end of phase 3 (November 2023), the abundance of archaea had increased to 11.84 ± 14.27%, while the abundance of bacteria reached 88.16 ± 14.27%. Similarly, in the Mesocosm A mesocosm samples at the end of phase 2, archaea and bacteria accounted for 31.54 ± 7.38% and 60.42 ± 7.72% of the taxa, respectively. By the end of phase 3 (November 2023), the abundance of archaea decreased to 5.86 ± 3.44%, and the abundance of bacteria increased to 94.14 ± 3.44%. The relatively lower abundance of archaea in Mesocosm B samples confirms the slower methane production process at the early stage of the monitoring process. Overall, the result indicates that chemical treatments were effective in both Mesocosm A and Mesocosm B samples to minimize the abundance of archaea.

Analysis of taxa at the phylum level (Figure S5) revealed that phyla including Spirochaetes, Firmicutes, Proteobacteria, Halobacteriota (archaea), or Chloroflexi were predominant in the samples in February 2023, independent of the origin of the tailings (Mesocosm B vs. Mesocosm A), the solvents (paraffinic vs. naphthenic), or the chemical treatments used. Most of the dominant taxa at the start of the treatment remained prevalent throughout, although a shift was observed at the end of the treatment. Meanwhile, a comparison of the dominant phyla at the end of phase 3 (November 2023), considering the type of tailings (Mesocosm B vs. Mesocosm A) and solvents (paraffinic vs. naphthenic) showed that the naphthenic Mesocosm B tailings was associated with a more diverse group of dominant phyla compared to the naphthenic Mesocosm A tailings. In contrast, a less diverse group of phyla was observed in the paraffinic Mesocosm B and Mesocosm A samples (Figure S5 and Table S3). Thus, the results indicate that the naphthenic diluent generally supports greater methanogen diversity than the paraffinic diluent. Paraffinic solvent systems more rapidly suppressed methanogenesis with Fe_2_(SO_4_)_3_, likely due to better support of Fe-reducers and sulfate-reducers.

A more detailed examination of the taxa at the family level at the end of phase 2 (February 2023) revealed the presence of three dominant (Relative Abundance, RA > 3%) families including Comamonadaceae (belonging to the phylum Proteobacteria), Anaerolineaceae (belonging to the phylum Chloroflexi), and Spirochaetaceae (belonging to the phylum Spirochaetota) in the Mesocosm B samples. In contrast, the Mesocosm A samples were dominated by members of Methanoregulaceae (belonging to the phylum Euryarchaeota), Desulfotomaculales (belonging to the phylum Firmicutes), and Anaerolineaceae (belonging to the phylum Chloroflexi) (Figure 10). It has been reported that the members of Desulfotomaculales are involved in the reduction of sulfate (SO_4_^2–^) to hydrogen sulfide (H_2_S). For example, *Desulfosporosinus* is capable of using As­(V), Fe­(III), sulfate, thiosulfate, and nitrate as respiratory electron acceptors, or *Desulfitobacterium* is known for its ability to reduce both Fe­(III) and SO_4_^2–^. Additionally, *Desulfosporosinus* can incompletely oxidize organic substrates, such as lactate, pyruvate, and ethanol, to acetate. Both *Desulfosporosinus* and *Desulfitobacterium* have been shown to decompose propionic acid, benzoic acid, acetic acid, and aromatic compounds.^32^ This indicates that the sulfate reducing process was more active for Mesocosm A than Mesocosm B in Feb 2023.

**Figure 10:**
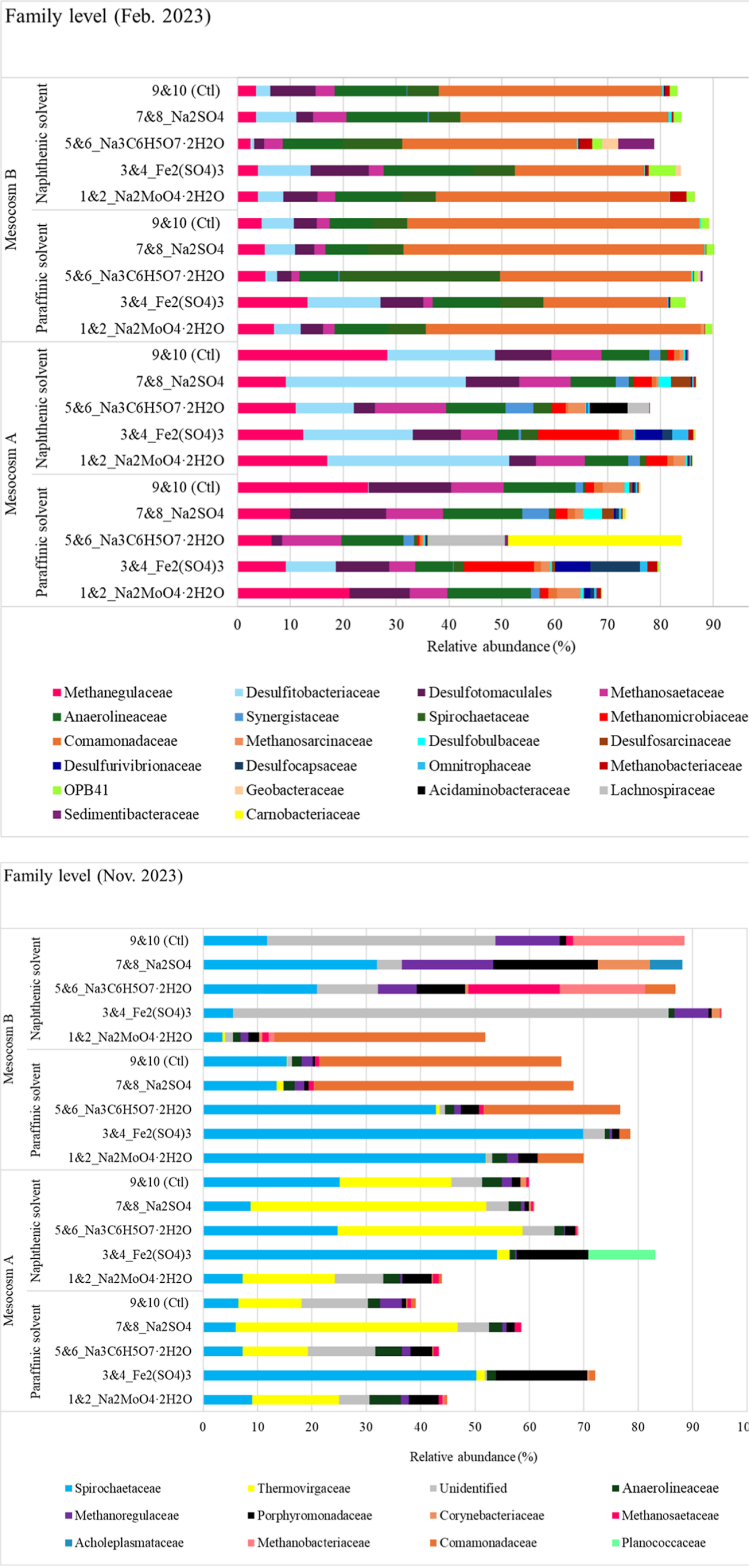
Family level microbial community profile for mesocosm tailings sampled in February 2023 (phase 2) and November 2023 (phase 3). Data for duplicated samples of each treatment condition were averaged.

A comparison of the taxa in the samples at the end of phase 3 (November 2023) with those in February 2023 showed a shift in the dominant taxa in both Mesocosm B and Mesocosm A after the chemical treatments (Na_2_MoO_4_·2H_2_O, Fe_2_(SO_4_)_3_, Na_3_C_6_H_5_O_7_·2H_2_O, Na_2_SO_4_, or control). Microbial diversity and methanogen abundance significantly declined by Nov 2023. This highlights the effect of solvent or chemical treatments on the microbial communities in the mesocosms. For example, while the members of the family Comamonadaceae were dominant in February 2023 in Mesocosm B samples, this family were replaced in November 2023 by the members of Spirochaetaceae, which were previously reported in oil-associated environments as some members are known to be involved in hydrocarbon-degradation methanogenic activity. Furthermore, it appears that Spirochaetaceae becomes dominant across nearly all samples in November 2023, indicating a possible environmental shift favoring this family. Meanwhile, samples in February 2023 contain more methanogenic families such as Methanosaetaceae, Methanomicrobiaceae, and Methanoregulaceae, suggesting active methane cycling; by November 2023, methanogen presence is reduced, with Methanobacteriaceae and Methanoregulaceae appearing in smaller proportions.

A quantitative comparison of methanogenic family abundance across treatments between February and November 2023 suggests varying degrees of methane inhibition by the tested chemical treatments. The relative abundance of key methanogenic families, including Methanosaetaceae, Methanobacteriaceae, Methanomicrobiaceae, and Methanoregulaceae, declined substantially over time in all treatments, with the extent of reduction serving as a proxy for methane inhibition. As shown in Table S2, among the treatments, Fe_2_(SO_4_)_3_ demonstrated the highest inhibition, with methanogen abundance decreasing from approximately 23% in February to below 1% in November. Na_2_MoO_4_·2H_2_O and Na_2_SO_4_ also exhibited some degree of inhibition, depending on the diluents or mesocosms. In contrast, Na_3_C_6_H_5_O_7_·2H_2_O showed a relatively higher persistence of methanogenic populations. A similar trend has been observed in both Mesocosm A and Mesocosm B, although methanogen inhibition was generally stronger in Mesocosm B, which may correlate with the delayed methane production process in Mesocosm B. These findings suggest that Fe_2_(SO_4_)_3_ and Na_2_MoO_4_·2H_2_O treatments are more effective in suppressing methanogenic activity over time, whereas citrate sustains or stimulates microbial communities that support methane production.

Analysis of the microbial community composition revealed the presence of iron-reducing bacterial families, particularly in the February 2023 samples (Figure 10). Geobacteraceae, a well-known group of dissimilatory Fe­(III) reducers, were most abundant in the Fe_2_(SO_4_)_3_-treated and citrate-treated samples. This suggests that the availability of ferric iron in the Fe_2_(SO_4_)_3_ treatment likely promoted the growth of *Geobacter* spp. However, by November 2023, the abundance of Geobacteraceae had declined significantly. The decline in the abundances of iron-reducing bacteria reflects the depletion of bioavailable ferric iron, a shift in redox conditions, or increased competition from other anaerobic functional groups such as Spirochaetaceae. These observations suggest that iron reduction was likely active in the early stages of mesocosm incubation but diminished over time as the environmental conditions evolved.

Shannon Diversity Index (*H* = (−∑*P*_*i*_ ln­(*P*_*i*_)), where *P*_*i*_ = proportion of class (i), was calculated (Figure 11) based on the taxa with RA > 3% to assess the effect of solvents or chemical treatments on microbial diversity. A comparison of the alpha diversity in February 2023 and at the end of the experimental period (November 2023) revealed changes in microbial diversity following the treatments. Overall, the alpha diversity decreased in all treatments at the end of the experimental period (November 2023). Diversities of 1.68 (*n* = 40) and 1.05 (*n* = 40) were observed in February 2023 and November 2023, respectively. At the end of the treatment period (November 2023), the lowest Shannon diversity was observed in the samples treated with Fe_2_(SO_4_)_3_.

**Figure 11:**
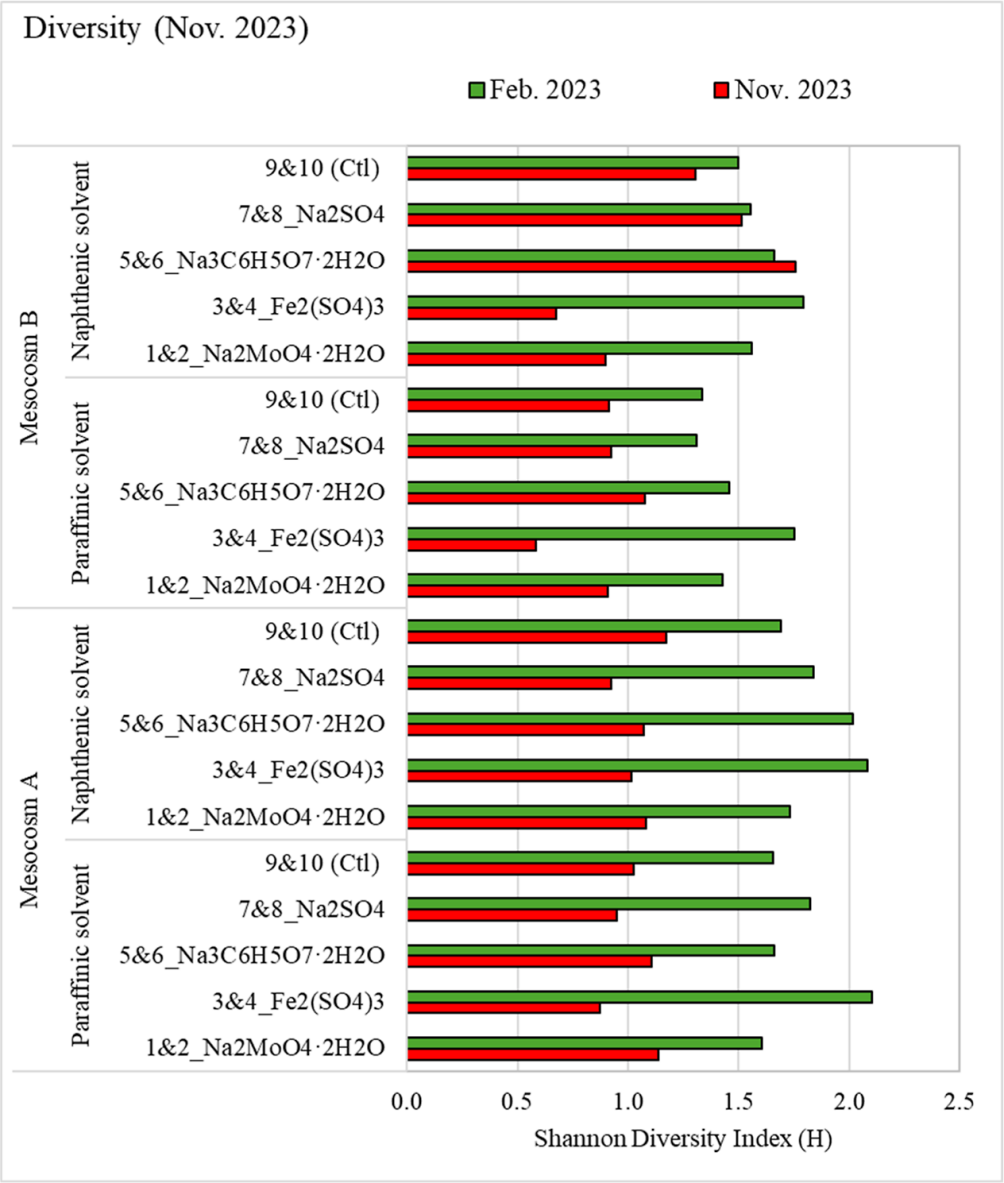
Changes in the Shannon diversity of dominant (RA > 3%) microbial taxa at the family level. Data for duplicated samples of each treatment condition were averaged.

Overall, the changes in the microbial community structure and diversity emphasize the impact of both solvents and chemicals on the microbial diversity and community structure in oil sands tailings. The Shannon diversity index analysis demonstrated a notable decrease in the level of microbial diversity following chemical treatments. Among the different treatments, Fe_2_(SO_4_)_3_ resulted in the lowest microbial diversity, while Na_3_C_6_H_5_O_7_·2H_2_O and Na_2_SO_4_ treatments yielded higher diversity levels in naphthenic Mesocosm B samples.

In addition, at the end of phase 2 (February 2023), active sulfate-reducing bacteria and iron-reducing bacteria likely inhibited methane production via competitive exclusion of methanogens for electron donors. Treatments with Fe_2_(SO_4_)_3_ showed the most pronounced effect, with elevated levels of Geobacteraceae and Desulfobulbaceae, indicating that both Fe­(III) and sulfate served as terminal electron acceptors in this phase. In phase 3 (November 2023), although methanogen abundance remained low, iron-reducing bacteria and sulfate-reducing bacteria populations also declined, suggesting that methane inhibition was maintained not by direct competition but by altered community structure, resource depletion, and possible accumulation of inhibitory byproducts. These findings support a dynamic inhibition model in which electron acceptor competition dominates initially, followed by ecological stabilization that disfavors methanogenesis. Mesocosm B shows stronger methane inhibition, likely due to delayed methane production. However, both systems reach a low-methane state by November due to longer-term ecological shifts. Despite substantial suppression of methanogenic families by Fe_2_(SO_4_)_3_ and Na_2_MoO_4_·2H_2_O, the persistence or resurgence of methane-producing populations under some treatments suggests that additional or sequential chemical treatments may be required to achieve sustained methane mitigation in oil sands tailings.

### Brief Cost Analysis

According to the IMARC group, a Market Research Firm, the price of ferric sulfate is between 209 and 329 USD per MT as of December 2023 and the price of sodium sulfate is between 216 and 460 USD per MT depending on the countries of the suppliers.^33,34^ While there is no publicly reported pricing for sodium molybdate, it is expected to be more expensive than sodium sulfate and ferric sulfate and, therefore, has not been included in the cost analysis in this study. As of 2023, tailings ponds in Alberta contained approximately 1.5 billion m^3^ of fluid tailings.^35^ And these treatment chemicals could be added into an active oil sands tailings pond through an in-line mixer with tailings streams. Since 6931.25 mg/L ferric sulfate and 5882.00 mg/L sodium sulfate were used to inhibit the methane emission in the mesocosm B slurry, we will use these concentrations to calculate how much chemicals would be needed for the entire fluid tailings in Alberta. The results show roughly 10.4 million metric tons of ferric sulfate and 8.82 million metric tons of sodium sulfate would be required for the initial tailings treatment. Given the price of the chemicals, the initial treatment cost would be about 2 to 4 billion USD. While substantial in cost, this tailing treatment represents an economically viable approach relative to other emerging or novel technologies while simultaneously delivering multiple key benefits such as methane reduction, tailings consolidation, water recovery, and risk mitigation. Therefore, it could represent a strategically viable and environmentally beneficial approach, especially for the active ponds, mitigating long-term risks and enabling land reclamation.

## Conclusions

This study demonstrates, through laboratory-scale experiments, the potential for using economically viable chemical treatments to reduce greenhouse gas (GHG) emissions from oil sands tailings ponds. It further highlights the interplay among water chemistry, microbial community dynamics, and GHG production. Among the three chemicals evaluated, Na_2_MoO_4_·2H_2_O, Fe_2_(SO_4_)_3_, and Na_2_SO_4_ all showed the ability to inhibit methanogens directly within the tailings matrix. Fe_2_(SO_4_)_3_, in particular, offered dual benefits by not only suppressing methane production but also promoting tailings consolidation. While the type of residual solvent, paraffinic or naphthenic, influenced the magnitude of methane emissions, the overall effectiveness of chemical inhibition remained consistent across solvent types. Nevertheless, this technology remains at an early stage as oil sands tailings present unique challenges due to their high salinity and recalcitrant organic content. Field-scale demonstrations in operational tailings ponds will be essential for advancing toward commercial application.

Microbial community profiling of the mesocosm study revealed significant shifts in both diversity and structure, shaped by the combined influence of solvent type and chemical treatment. Bacteria consistently outnumbered archaea, with bacterial abundance increasing by the end of the treatment period, while archaeal populations including methanogens declined. Taxonomic analyses identified dominant phyla, such as Spirochaetota, Firmicutes, Proteobacteria, and Halobacteriota, with dynamic changes in their relative abundances linked to specific treatments. Naphthenic Mesocosm B exhibited greater taxonomic diversity at the phylum level, while paraffinic samples showed reduced diversity. At the family level, early microbial communities were dominated by Comamonadaceae and Methanoregulaceae, which were later replaced by Spirochaetaceae and Thermovirgaceae, particularly in response to chemical treatments. The Shannon diversity index suggested Fe_2_(SO_4_)_3_ treatments led to a marked reduction in microbial diversity, likely due to the selective stimulation of sulfate- and iron-reducing bacteria. These findings highlight the significant influence of both solvent type and chemical treatment on microbial ecology in tailings systems and suggest that these factors are key levers in managing biogenic methane emissions in oil sands environments.

## Supplementary Material



## Notes

Experimental schematic flowchart; temporal evolution of methane production in phase 1; changes in the abundance of archaea and bacteria over the experimental period; dominant phyla in the mesocosms; and number of bottle samples at different conditions (PDF)
