# Supplementary material for: Mesocosm Study of Chemical Treatments on Methane Emissions in Oil Sands Tailings Ponds—Part I: Focusing on the Change of Microbial Communities and Tailings Dewaterability
Source: ACS Omega. 2025 Oct 13;10(41):48948–64. doi: 10.1021/acsomega.5c07316 (PMC12547779; doi:10.1021/acsomega.5c07316)
Supplement: Supplementary file 1 [file ao5c07316_si_001.pdf]

Supplementary Information for:

Mesocosm Study of Chemical Treatments on Methane Emissions in Oil Sands Tailings Ponds – Part I: Focusing on the Change of Microbial Communities and Tailings Dewaterability

Xiaomeng Wang[1], Nayereh Saborimanesh[1], Petr Kuznetsov[2], Amanda Cook[1], Jordan Elias[1], Louis Jugnia[3], Bipro Ranjan Dhar[2] and Ania Ulrich[2]*

[1]Natural Resources Canada, CanmetENERGY Devon, 1 Oil Patch Drive, Devon, Alberta T9G 1A8, Canada

[2]Faculty of Engineering, Civil and Environmental Engineering Department, University of Alberta, 9211 116 Street, Edmonton, Alberta, T6G 2H5, Canada

[3]Energy, Mining and Environment Research Centre, National Research Council Canada, 6100 Royalmount Avenue, Montreal, Quebec H4P 2R2, Canada

*Corresponding Author: xiaomeng.wang@nrcan-rncan.gc.ca

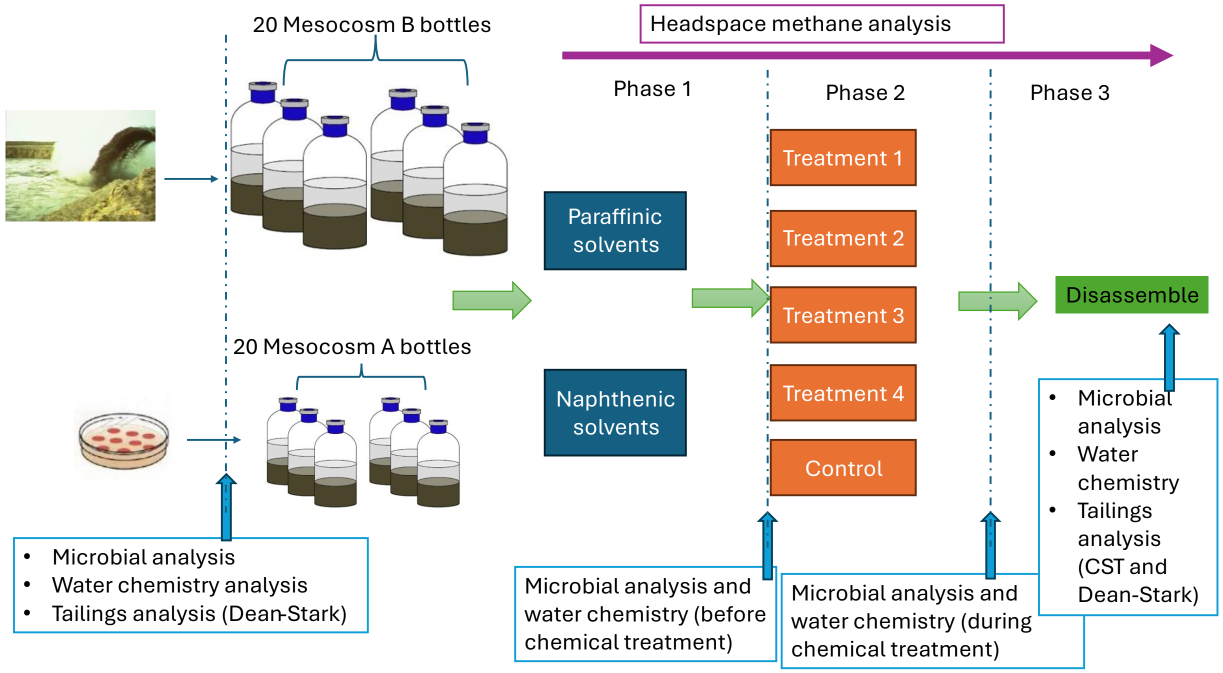

Figure S1 – Experimental schematic flowchart illustrating the process from sample preparation through chemical addition to sampling for different analyses; CST is capillary suction time.

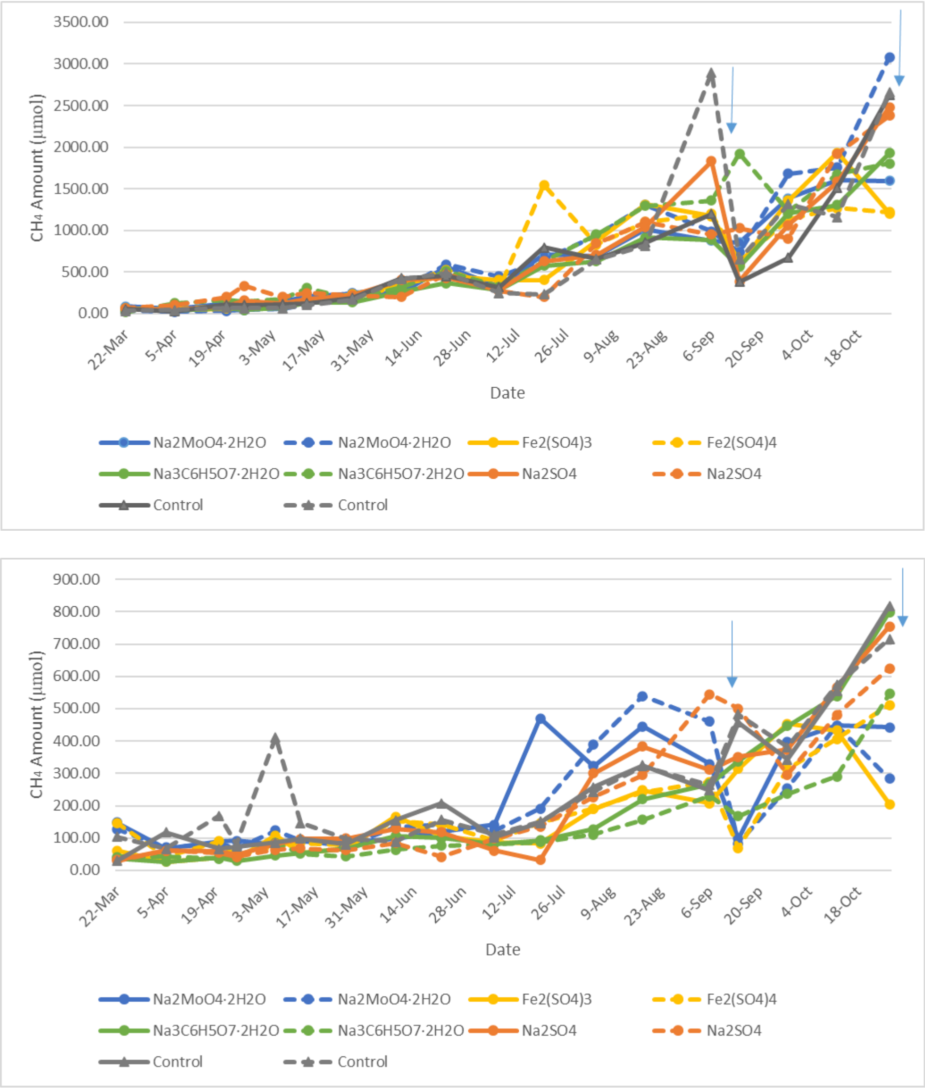

Figure S2 – Temporal evolution of methane production in the headspace of the Mesocosm A naphthenic bottles (top) and Mesocosm A paraffinic bottles (bottom) in phase 1; treatments are shown as colored dotted lines, with duplicates represented by solid and dashed lines of the same color; arrows show the chemical addition time points.

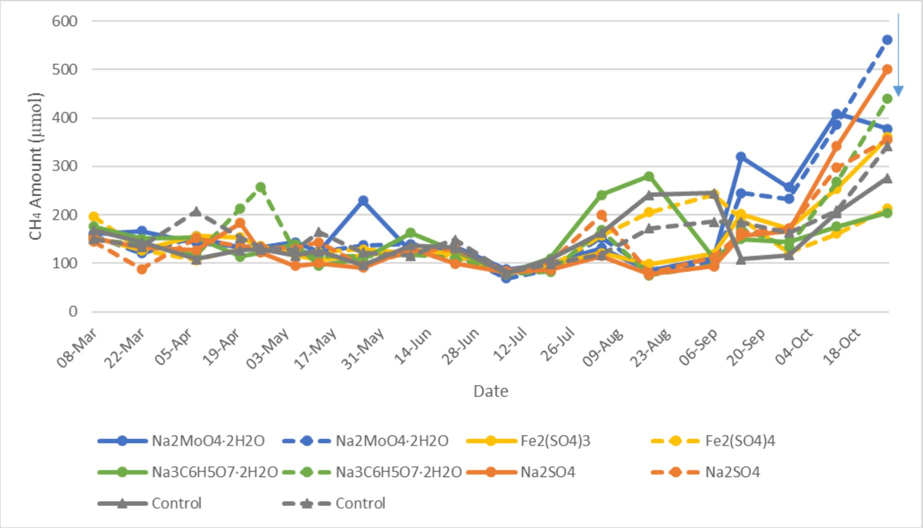

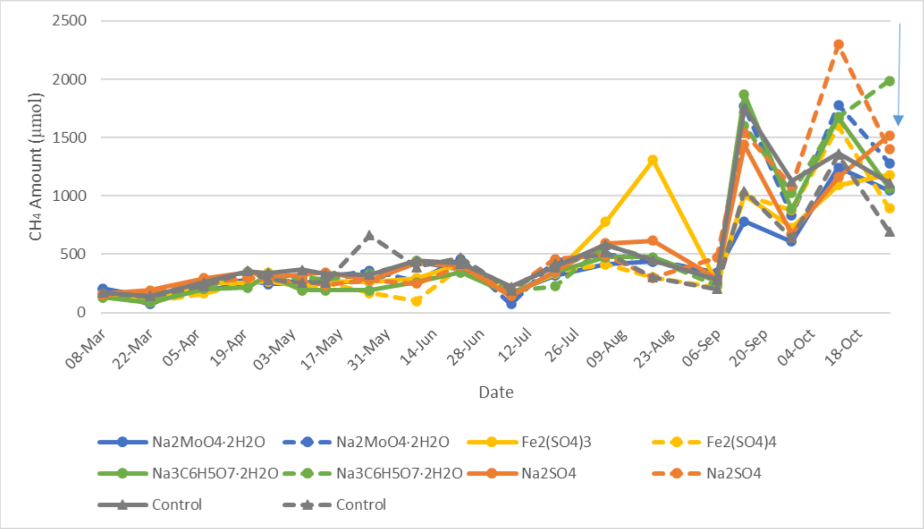

Figure S3 – Temporal evolution of methane production in the headspace of the Mesocosm B naphthenic bottles (top) and Mesocosm B paraffinic bottles (bottom) in phase 1; treatments are shown as colored dotted lines, with duplicates represented by solid and dashed lines of the same color; arrow shows the chemical addition time point.

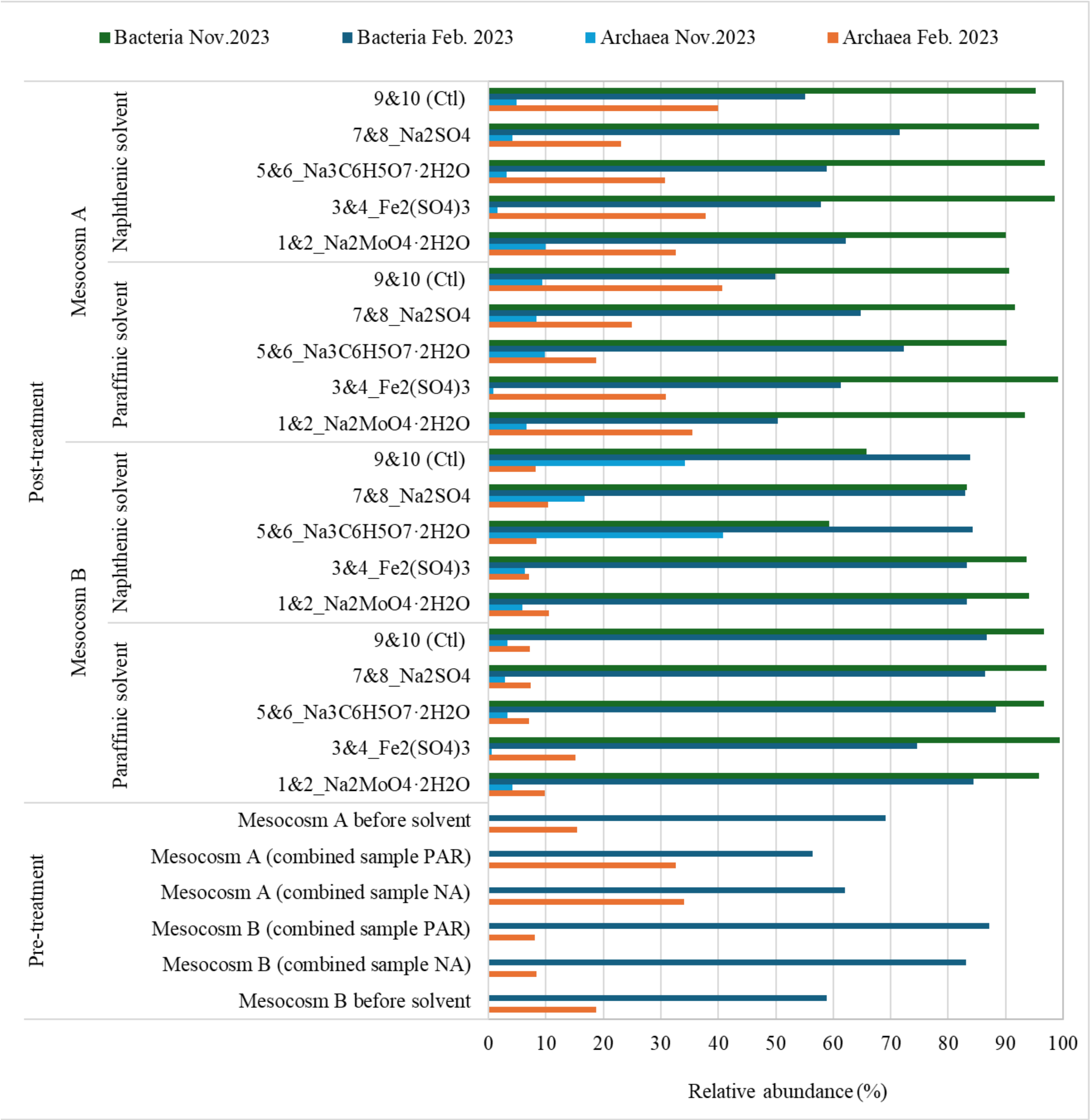

Bacteria Nov.2023
Bacteria Feb. 2023
Archaea Nov.2023
Archaea Feb. 2023
Post-treatment
Mesocosm A
Naphthenic solvent
Paraffinic solvent
Mesocosm B
Pre-treatment
9&10 (Ctl)
7&8_Na2SO4
5&6_Na3C6H5O7·2H2O
3&4_Fe2(SO4)3
1&2_Na2MoO4·2H2O
Mesocosm A before solvent
Mesocosm A (combined sample PAR)
Mesocosm A (combined sample NA)
Mesocosm B (combined sample PAR)
Mesocosm B (combined sample NA)
Mesocosm B before solvent
0 10 20 30 40 50 60 70 80 90 100
Relative abundance (%)

Figure S4 – Changes in the abundances of archaea and bacteria over the experimental period. Feb. 2023 is at the end of phase 2 and Nov. 2023 represents the end of phase 3. Pre-treatment samples including before-solvent addition samples named as Mesocosm A & B before solvent, and post-solvent addition samples named as Mesocosm A & B (combined sample PAR and NA). PAR represents paraffinic solvents; NA represents naphthenic solvents. Numbers represent duplicate treatments; Ctl indicates control samples.

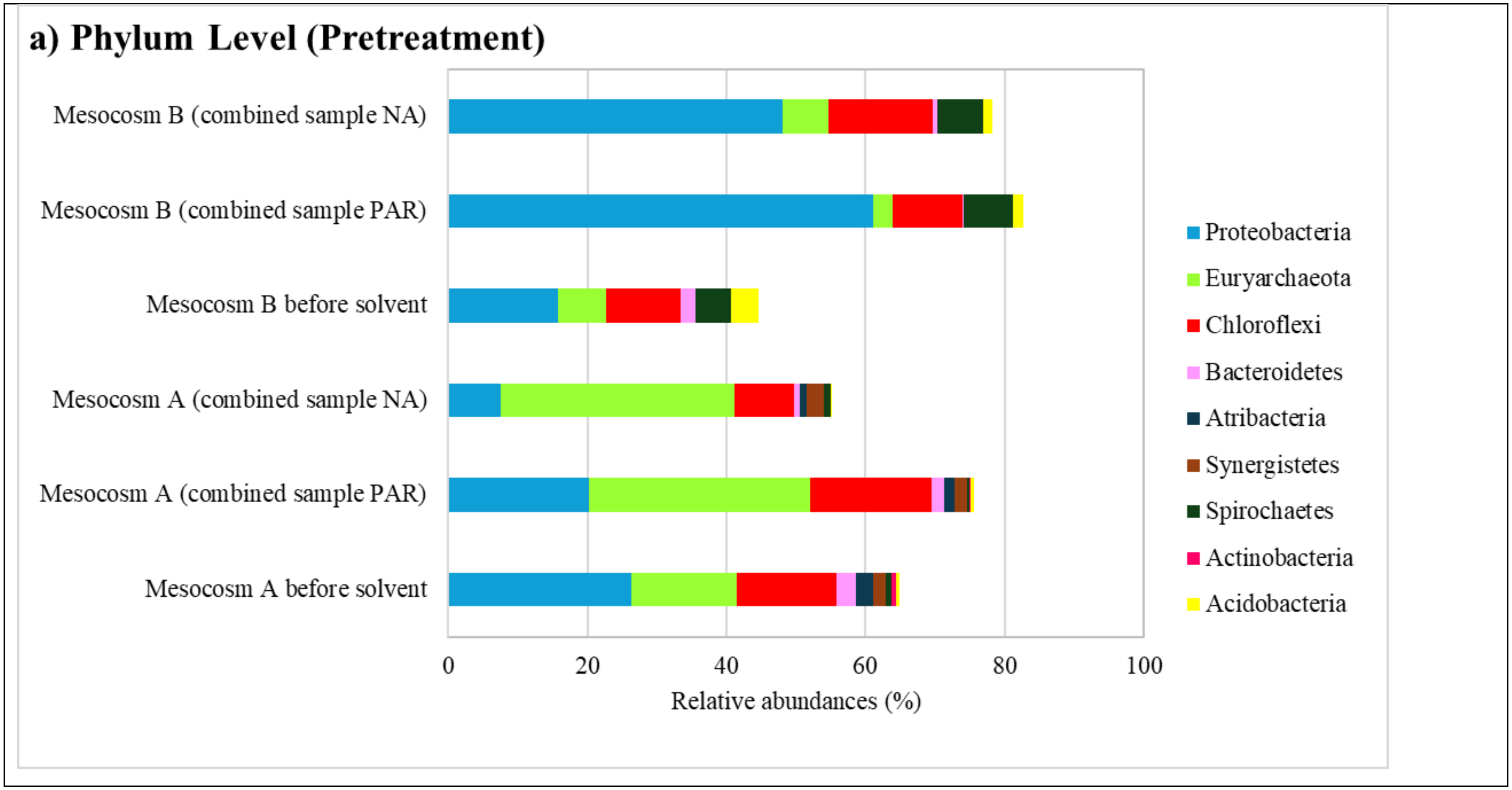
a) Phylum Level (Pretreatment)
Mesocosm B (combined sample NA)
Mesocosm B (combined sample PAR)
Mesocosm B before solvent
Mesocosm A (combined sample NA)
Mesocosm A (combined sample PAR)
Mesocosm A before solvent
0
20
40
60
80
100
Relative abundances (%)
Proteobacteria
Euryarchaeota
Chloroflexi
Bacteroidetes
Atribacteria
Synergistetes
Spirochaetes
Actinobacteria
Acidobacteria

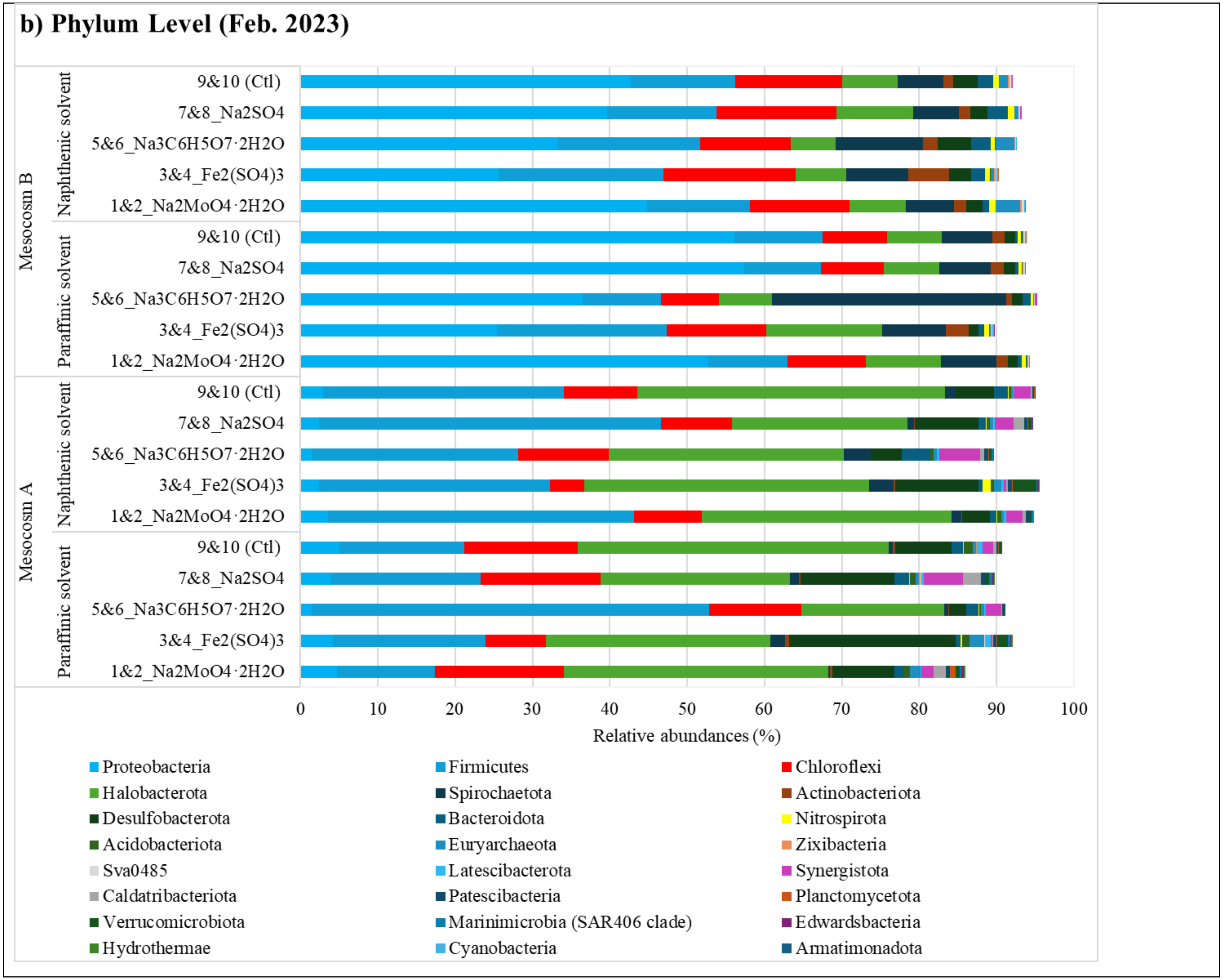
b) Phylum Level (Feb. 2023)
Mesocosm B
Naphthenic solvent
Paraffinic solvent
Mesocosm A
Naphthenic solvent
Paraffinic solvent
9&10 (Ctl)
7&8_Na2SO4
5&6_Na3C6H5O7·2H2O
3&4_Fe2(SO4)3
1&2_Na2MoO4·2H2O
0
10
20
30
40
50
60
70
80
90
100
Relative abundances (%)
Proteobacteria
Firmicutes
Chloroflexi
Halobacterota
Spirochaetota
Actinobacteriota
Desulfobacterota
Bacteroidota
Nitrospirota
Acidobacteriota
Euryarchaeota
Zixibacteria
Sva0485
Latescibacterota
Synergistota
Caldatribacteriota
Patescibacteria
Planctomycetota
Verrucomicrobiota
Marinimicrobia (SAR406 clade)
Edwardsbacteria
Hydrothermae
Cyanobacteria
Armatimonadota

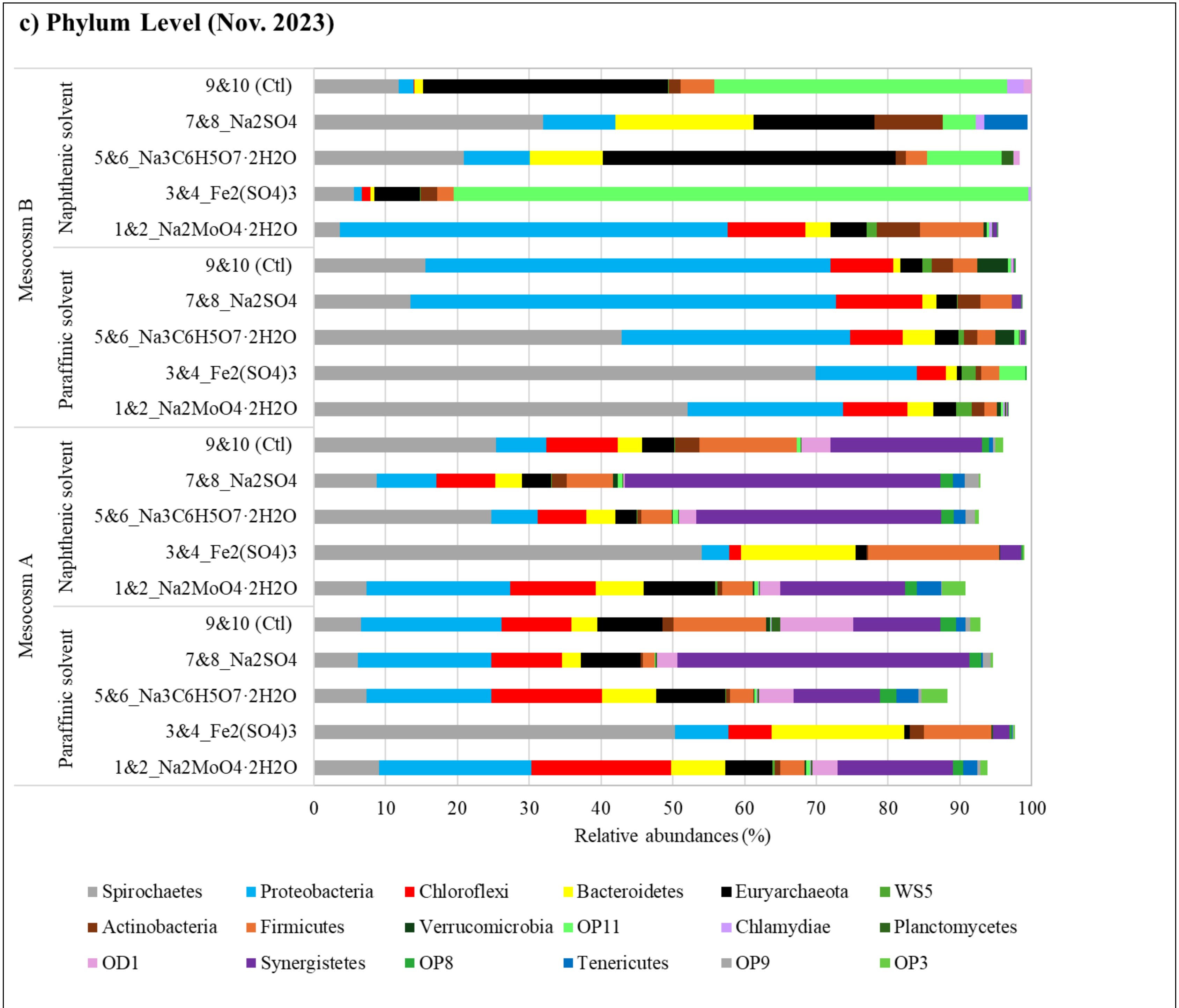
c) Phylum Level (Nov. 2023)
Mesocosm B
Mesocosm A
Naphthenic solvent
Paraffinic solvent
9&10 (Ctl)
7&8_Na2SO4
5&6_Na3C6H5O7·2H2O
3&4_Fe2(SO4)3
1&2_Na2MoO4·2H2O
0
10
20
30
40
50
60
70
80
90
100
Relative abundances (%)
Spirochaetes
Proteobacteria
Chloroflexi
Bacteroidetes
Euryarchaeota
WS5
Actinobacteria
Firmicutes
Verrucomicrobia
OP11
Chlamydiae
Planctomycetes
OD1
Synergistetes
OP8
Tenericutes
OP9
OP3

Figure S5. Dominant phyla with relative abundances above 1% in the tailings at the start of the experiment before chemical treatment (a), at the end of phase 2 (Feb. 2023) (b), and at the end of phase 3 (Nov 2023) (c). Before chemical treatment samples includes before-solvent addition samples named as Mesocosm A & B before solvent, and post-solvent addition samples named as Mesocosm A & B (combined sample PAR and NA). PAR represents paraffinic solvents; NA represents naphthenic solvents. Numbers represent duplicate treatments; Ctl indicates control samples.

Tables

Table S1. Number of bottle samples at different conditions

# of Samples	*Mesocosm A*		*Mesocosm B*	
	Naphthenic	Paraffinic	Naphthenic	Paraffinic
$Na_2MoO_4{\cdot}2H_2O$	2	2	2	2
$Fe_2(SO_4)_3$	2	2	2	2
$Na_3C_6H_5O_7{\cdot}2H_2O$	2	2	2	2
Na_2SO_4	2	2	2	2
Control	2	2	2	2

Table S2. The changes in the abundance of archaea and bacteria in the pre-solvent sample, post-solvent samples, at the end of phase 2 (Feb. 2023) and at the end of phase 3 (Nov 2023).

	Mesocosm A (averaged treatments)				Mesocosm B (averaged treatments)			
	Pre-solvent (n=1)	Post-solvent (n=2)	At the end of phase 2 (n=20)	At the end of phase 3 (n=20)	Pre-solvent (n=1)	Post-solvent (n=2)	At the end of phase 2 (n=20)	At the end of phase 3 (n=20)
Archaea	15.50	33.30 ± 0.99	31.54 ± 7.38	5.86 ± 3.44	18.80	8.25 ± 0.20	9.09 ± 2.51	11.84 ± 14.27
Bacteria	69.10	59.25 ± 2.00	60.42 ± 7.72	94.14 ± 3.44	58.90	83.10 ± 2.90	83.79 ± 3.66	88.16 ± 14.27

	Averaged Mesocosm A and B in both solvents											
	Pre-solvent	Post-solvent	At the end of phase 2 (23-Feb)					At the end of phase 3 (23-Nov)				
	(n=2)	(n=4)	*$Na_2MoO_4{\cdot}2H_2O$* (n=8)	*$Fe_2(SO_4)_3$* (n=8)	*$Na_3C_6H_5O_7{\cdot}2H_2O$* (n=8)	*Na_2SO_4* (n=8)	*Control* (n=8)	*$Na_2MoO_4{\cdot}2H_2O$* (n=8)	*$Fe_2(SO_4)_3$* (n=8)	*$Na_3C_6H_5O_7{\cdot}2H_2O$* (n=8)	*Na_2SO_4* (n=8)	*Control* (n=8)
Archaea	17.15	20.78	22.68	23.00	12.90	16.13	23.95	5.40	0.73	6.62	5.60	6.33
Bacteria	64.00	71.18	67.43	67.95	80.30	75.63	68.30	94.61	99.27	93.39	94.41	93.67

Table S3. The three most dominant phyla at the end of phase 2 (Feb. 2023) and at the end of phase 3 (Nov 2023).

Type of FFT	Solvents	Treatments	Top Three Phyla	
			Phase 2 (Feb. 2023)	Phase 3 (Nov. 2023)
Mesocosm B	Paraffinic	$Na_2MoO_4 \cdot 2H_2O$	Proteobacteria;Firmicutes;Chloroflexi	Spirochaetes;Proteobacteria;Chloroflexi
		$Fe_2(SO_4)_3$	Proteobacteria;Firmicutes;Halobacterota	Spirochaetes;Proteobacteria;Chloroflexi
		$Na_3C_6H_5O_7 \cdot 2H_2O$	Proteobacteria;Spirochaetota;Firmicutes	Spirochaetes;Proteobacteria;Chloroflexi
		Na_2SO_4	Proteobacteria;Firmicutes;Chloroflexi	Proteobacteria;Spirochaetes;Chloroflexi
		Control	Proteobacteria;Firmicutes;Chloroflexi	Proteobacteria;Spirochaetes;Chloroflexi
	Naphthenic	$Na_2MoO_4 \cdot 2H_2O$	Proteobacteria;Firmicutes;Chloroflexi	Proteobacteria;Chloroflexi;Firmicutes
		$Fe_2(SO_4)_3$	Proteobacteria;Firmicutes;Chloroflexi	OP11;Euryarchaeota;Spirochaetes
		$Na_3C_6H_5O_7 \cdot 2H_2O$	Proteobacteria;Firmicutes;Chloroflexi	Euryarchaeota;Spirochaetes;OP11
		Na_2SO_4	Proteobacteria;Chloroflexi;Firmicutes	Spirochaetes;Bacteroidetes;Euryarchaeota
		Control	Proteobacteria;Chloroflexi;Firmicutes	OP11;Euryarchaeota;Spirochaetes
Mesocosm A	Paraffinic	$Na_2MoO_4 \cdot 2H_2O$	Halobacterota;Chloroflexi;Firmicutes	Proteobacteria;Chloroflexi;Synergistetes
		$Fe_2(SO_4)_3$	Halobacterota;Desulfobacterota;Firmicutes	Spirochaetes;Bacteroidetes;Firmicutes
		$Na_3C_6H_5O_7 \cdot 2H_2O$	Firmicutes;Halobacterota;Chloroflexi	Proteobacteria;Chloroflexi;Synergistetes
		Na_2SO_4	Halobacterota;Firmicutes;Chloroflexi	Synergistetes;Proteobacteria;Chloroflexi
		Control	Halobacterota;Firmicutes;Chloroflexi	Proteobacteria;Firmicutes;Synergistetes
	Naphthenic	$Na_2MoO_4 \cdot 2H_2O$	Firmicutes;Halobacterota;Chloroflexi	Proteobacteria;Synergistetes;Chloroflexi
		$Fe_2(SO_4)_3$	Halobacterota;Firmicutes;Desulfobacterota	Spirochaetes;Firmicutes;Bacteroidetes

		$Na_3C_6H_5O_7·2H_2O$	Halobacterota;Firmicutes;Chloroflexi	Synergistetes;Spirochaetes;Chloroflexi
		Na_2SO_4	Firmicutes;Halobacterota;Chloroflexi	Synergistetes;Spirochaetes;Proteobacteria
		Control	Halobacterota;Firmicutes;Chloroflexi	Spirochaetes;Synergistetes;Firmicutes
